# High burden of viruses and bacterial pathobionts drives heightened nasal innate immunity in children

**DOI:** 10.1084/jem.20230911

**Published:** 2024-07-01

**Authors:** Timothy A. Watkins, Alex B. Green, Julien A.R. Amat, Nagarjuna R. Cheemarla, Katrin Hänsel, Richard Lozano, Sarah N. Dudgeon, Gregory Germain, Marie L. Landry, Wade L. Schulz, Ellen F. Foxman

**Affiliations:** 1 Department of Laboratory Medicine, Yale School of Medicine, New Haven, CT, USA; 2 Department of Immunobiology, Yale School of Medicine, New Haven, CT, USA; 3 Department of Pediatrics, Yale School of Medicine, New Haven, CT, USA; 4 Department of Medicine, Yale School of Medicine, New Haven, CT, USA

## Abstract

Studies during the COVID-19 pandemic showed that children had heightened nasal innate immune responses compared with adults. To evaluate the role of nasal viruses and bacteria in driving these responses, we performed cytokine profiling and comprehensive, symptom-agnostic testing for respiratory viruses and bacterial pathobionts in nasopharyngeal samples from children tested for SARS-CoV-2 in 2021–22 (*n* = 467). Respiratory viruses and/or pathobionts were highly prevalent (82% of symptomatic and 30% asymptomatic children; 90 and 49% for children <5 years). Virus detection and load correlated with the nasal interferon response biomarker CXCL10, and the previously reported discrepancy between SARS-CoV-2 viral load and nasal interferon response was explained by viral coinfections. Bacterial pathobionts correlated with a distinct proinflammatory response with elevated IL-1β and TNF but not CXCL10. Furthermore, paired samples from healthy 1-year-olds collected 1–2 wk apart revealed frequent respiratory virus acquisition or clearance, with mucosal immunophenotype changing in parallel. These findings reveal that frequent, dynamic host–pathogen interactions drive nasal innate immune activation in children.

## Teaser

Children being tested for SARS-CoV-2 show heightened nasal innate immune responses compared with adults. Here, comprehensive PCR testing and cytokine profiling reveal that nasal respiratory viruses and bacterial pathobionts are highly prevalent in children and drive heightened nasal innate immunity.

## Graphical Abstract

**Figure:**
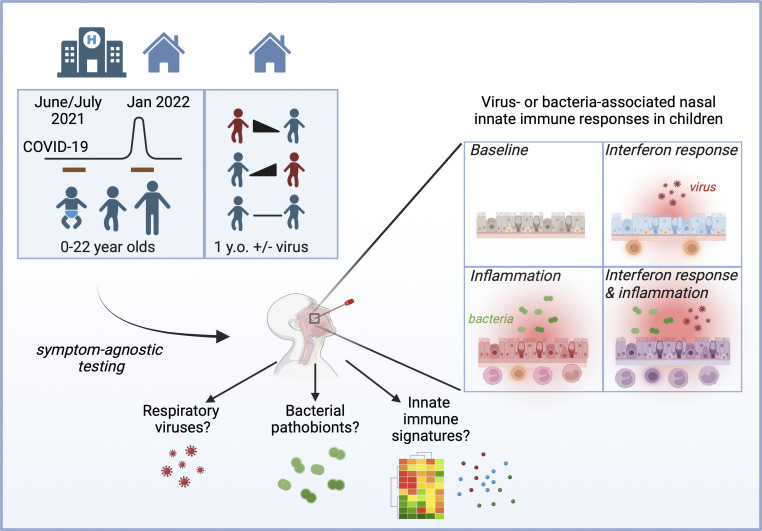

## Introduction

A puzzling feature of the COVID-19 pandemic has been its lower impact on children compared with adults, prompting a search for unique features of antiviral immunity in the pediatric age group (Kang and Jung, 2020). Recently, several independent studies found heightened nasal innate immune activation in children compared with adults with SARS-CoV-2 infection and even in the absence of SARS-CoV-2 infection (Loske et al., 2022; Mick et al., 2022; Pierce et al., 2021; Wimmers et al., 2023; Winkley et al., 2021; Yoshida et al., 2022). Although there was variability among subjects, nasal transcriptome patterns in children showed heightened expression of both interferon-stimulated genes (ISGs) and proinflammatory cytokines. Children also had an increased presence of leukocytes in the nasal mucosa including neutrophils, proinflammatory monocytes, and other cell types involved in innate and adaptive antiviral responses (e.g., NK cells, B cells, T cells). Since early SARS-CoV-2 replication relies on evasion of the interferon response within its target tissue (Minkoff and tenOever, 2023; Park and Iwasaki, 2020), preactivated nasal innate immunity in children has been proposed as a mechanism of protection from SARS-CoV-2. However, the biological drivers of heightened nasal innate immunity in children are unknown. It is also unclear whether there are distinct patterns of heightened nasal innate immunity with different drivers and functional consequences.

While it is possible that heightened nasal innate immunity in children is due solely to age-intrinsic biological mechanisms, these patterns could also result from a combination of age-intrinsic differences and dynamic mucosal immune responses to the high burden of viral and microbial mucosal infections in children. Pre-pandemic data show that children have higher rates of colonization with airway bacterial pathobionts and more frequent infections with common cold–causing respiratory viruses than adults, likely due to less well-developed adaptive immunity (Lambert and Culley, 2017; Toivonen et al., 2019). Recent epidemiological studies using multiplex virus detection have found surprisingly high rates of viral respiratory infections in asymptomatic children (Byington et al., 2015; Costa-Martins et al., 2021). Most studies describing viral or bacterial burden have not simultaneously evaluated nasal host responses to identified infections, but those that have suggest that even asymptomatic virus infection or pathobiont colonization can provoke a host mucosal cytokine and/or transcriptional response (Costa-Martins et al., 2021; Folsgaard et al., 2013; Jartti et al., 2008; Lambert and Culley, 2017; Wesolowska-Andersen et al., 2017; Wolsk et al., 2016; Yu et al., 2019). These observations suggest that high burdens of viruses and bacterial pathobionts in the airway may be central drivers of the heightened nasal innate immunity seen in children. If this model is correct, the presence and load of viruses and bacterial pathobionts at the time of sampling would be expected to predict the variable nasal immunophenotypes seen within the pediatric age group.

During 2021–2022, in our healthcare system, children with and without respiratory symptoms were screened for SARS-CoV-2 by reverse transcription quantitative PCR (RT-qPCR) testing of nasopharyngeal (NP) swabs prior to elective surgery and during evaluation in the emergency department (ED) for respiratory and non-respiratory illnesses. Curating residual swabs from these screening tests enabled us to test the relationship between NP immunophenotypes, viruses, and bacteria in children at times when different viruses were circulating. We focused our analysis on samples collected from June 3 to July 2, 2021, a timeframe when there were relatively few SARS-CoV-2 infections in children, and January 11–23, 2022, corresponding to the Omicron variant surge which had the highest incidence rate of COVID-19 cases in the U.S. to date, including pediatric cases (Centers for Disease Control, 2024; The New York Times, 2021).

Here, we report the results of comprehensive testing for 16 respiratory viruses and 3 bacterial pathobionts in these samples together with quantitative microfluidics-based assays for cytokines implicated in antiviral and antibacterial innate immune responses. By testing whether variation in nasal cytokine profile correlates with virus or pathobiont detection and load, we demonstrate distinct patterns of innate immune activation in pediatric subjects predicted by the types and burden of microbes present in the nasopharynx. Analysis of RNA sequencing (RNA-seq) data also links pathobionts to enhanced leukocyte infiltration of the nasal mucosa. Finally, analysis from paired longitudinal samples from healthy 1-year-olds showed that nasal respiratory viruses are common in healthy subjects and that nasal immunophenotype changes in parallel with viral acquisition or clearance in the same individual. Together, these results point to a high burden of respiratory viruses and bacterial pathobionts as key drivers of heightened nasal mucosal innate immunity in children.

## Results

### Comprehensive testing reveals high rates of nasal respiratory viruses and bacterial pathobionts in children in June/July 2021 and January 2022

**Figure 1.**
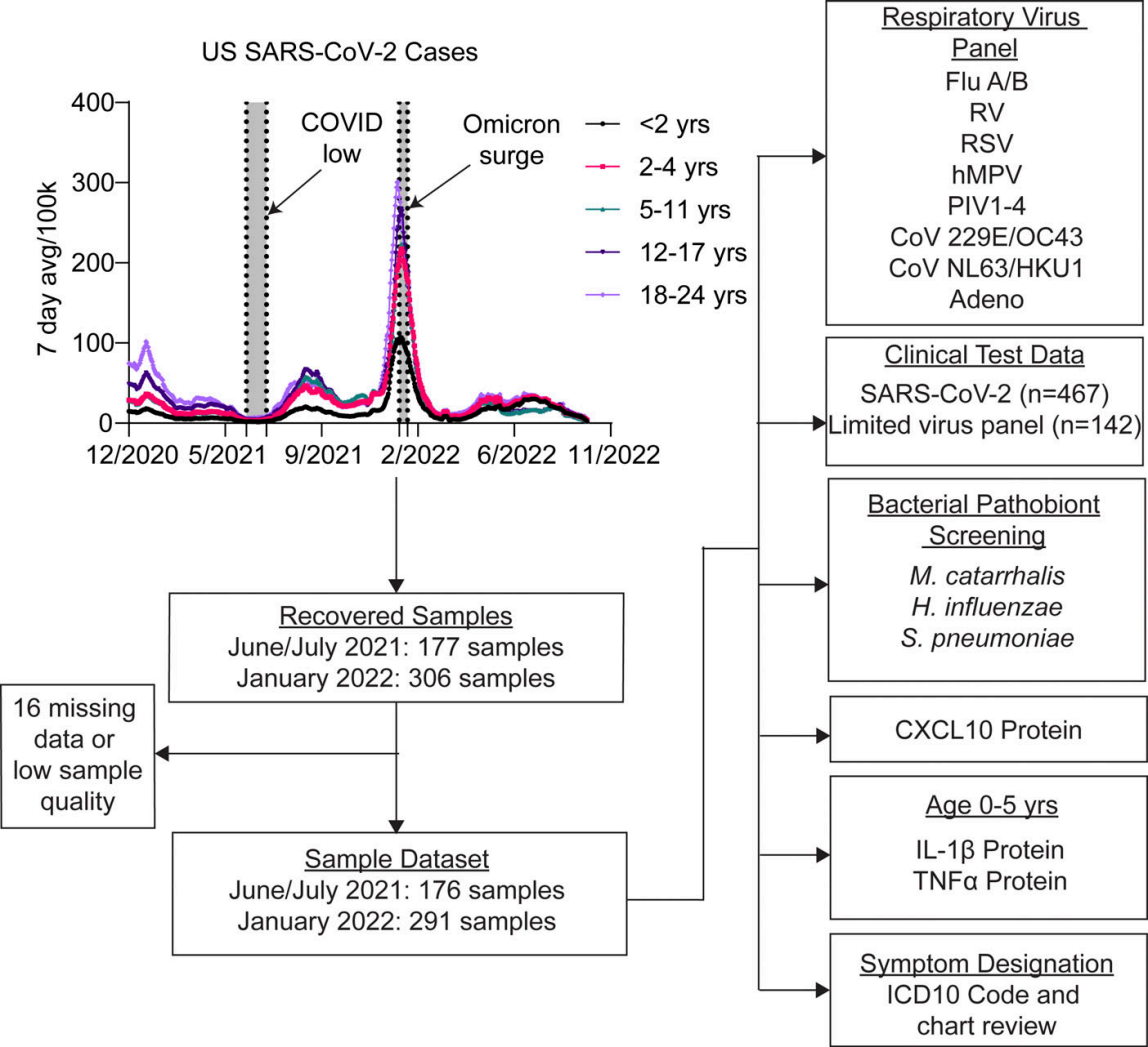
**Study overview.** NP swabs from subjects aged 0–22 undergoing SARS-CoV-2 testing in the YNHH pediatric ED or as part of preoperative screening were collected from June 3 to July 2, 2021, and January 11 to 23, 2022 corresponding to SARS-CoV-2 low and high circulation respectively. Samples were processed and analyzed as indicated for this study.

To better understand the relationship between mucosal innate immunity in pediatric subjects and the presence of respiratory viruses and bacterial pathobionts, we collected NP swab samples from subjects aged 0–22 years undergoing SARS-CoV-2 testing at the Yale New Haven Hospital (YNHH) Clinical Virology Laboratory from June 3 to July 2, 2021, a time period with relatively few cases of SARS-CoV-2 infection in children, and January 11–23, 2022 corresponding to the Omicron surge in the U.S. and highest rate of documented pediatric SARS-CoV-2 infections to date (Fig. 1 and Table S1).

**Figure 2.**
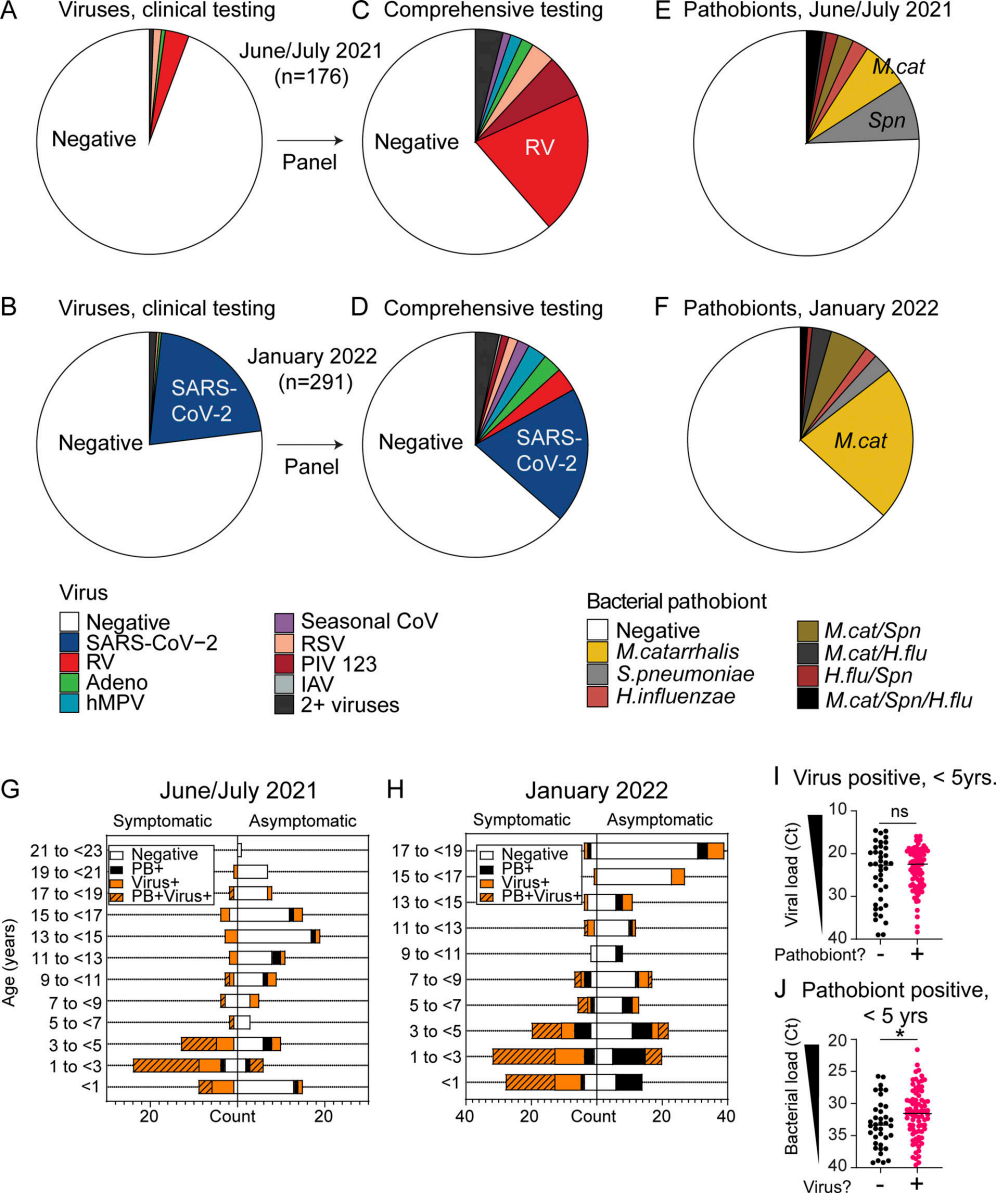
**Respiratory virus and bacterial pathobiont testing results. (A and B)** NP swab sample respiratory virus test results from pediatric ED clinical care testing from June 3 to July 2, 2021 (*n* = 176) (A) and January 11 to 23, 2022 (*n* = 291) (B). **(C and D)** NP swab sample respiratory virus test results following comprehensive 16-virus panel testing from June 3 to July 2, 2021 (*n* = 176) (C) and January 11 to 23, 2022 (*n* = 291) (D). **(E and F)** NP swab sample bacterial pathobiont testing results from June 3 to July 2, 2021 samples (*n* = 176) (E) and January 11 to 23, 2022 samples (*n* = 291) (F). **(G and H)** Number of samples testing negative for respiratory viruses and pathobionts (white), positive for respiratory viruses (orange), bacterial pathobionts (black), or both (orange/black pattern) as represented by subject age and clinical presentation for June 3–July 2, 2021 (*n* = 176) (G) and January 11–23, 2022 (*n* = 291) (H). **(I)** Viral load (represented as PCR Ct) within virus-positive samples from children <5 years old (*n* = 120) based on whether a bacterial pathobiont was detected. The Mann–Whitney test is used to compare viral load between groups. ns = not significant. **(J)** Pathobiont load (represented as Ct) within pathobiont-positive samples from children <5 years old (*n* = 118) based on whether a respiratory virus was detected. The Mann–Whitney test is used to compare pathobiont load between groups. *P = 0.0259.

Samples were from asymptomatic children undergoing preoperative screening for elective surgery and children presenting to the pediatric ED with symptoms of acute respiratory illness or for other reasons. Symptomatic or asymptomatic status was defined based on ICD-10 codes and chart review (Table S2). All available samples from June 3 to July 2, 2021 (*n* = 176) and January 11–23, 2022 (*n* = 291) were included (Fig. 1). Along with SARS-CoV-2, a subset of samples had been tested for additional respiratory viruses during patient care (*n* = 30 in June/July 2021; *n* = 112 in January 2022). Of the June/July 2021 samples, 10 tested positive for non-SARS-CoV-2 viruses during clinical care and none were positive for SARS-CoV-2 (Fig. 2 A). Of the January 2022 samples, 5 tested positive for non-SARS-CoV-2 viruses and 65 were positive for SARS-CoV-2 (Fig. 2 B). Of the 65 SARS-CoV-2^+^ subjects, 16 were asymptomatic, 34 were treated as outpatients, and 15 children were admitted to the hospital. Of these 15 admissions, 10 were attributable to comorbidities while 5 were for virus infection–related symptoms (Table S3).

To evaluate the presence of respiratory viruses that were not diagnosed during SARS-CoV-2 screening or patient care, all residual NP swabs (*n* = 467) were tested for 16 total respiratory viruses using a clinically validated panel of virus-specific quantitative PCR (qPCR) tests as described previously (Cheemarla et al., 2023a). Results revealed a much higher prevalence of respiratory viruses than what was diagnosed at the time of clinical testing. Rhinovirus (RV) predominated in June/July 2021 (43/176, 24.4% of samples) and SARS-CoV-2 predominated in January 2022 (65/291, 22.3% of samples), but many samples were positive for other seasonal respiratory viruses also in each timeframe (25/176 and 41/291), with overall virus positivity rates of 38.6 and 36.4% in June/July 2021 and January 2022, respectively (Fig. 2, C and D; and Table S4).

Next, we performed qPCR for the three most common bacterial pathobionts known to colonize the upper respiratory tract and contribute to clinically significant respiratory tract viral–bacterial coinfections: *Moraxella catarrhalis*, *Streptococcus pneumoniae*, and *Hemophilus influenzae* (Bogaert et al., 2004; Hendley et al., 2005; Murphy et al., 2009; Murphy and Parameswaran, 2009). All pathobionts were detected, with *M*. *catarrhalis* and *S*. *pneumoniae* being most common in June/July 2021 and *M. catarrhalis* being the most prevalent in January 2022 (Fig. 2, E and F; and Table S5). Overall, the pathobiont positivity rate was 24.4 and 36.9% in June/July 2021 and January 2022, respectively.

### Nasal viruses, bacterial pathobionts, or both were detected in the majority of children under 5 years of age

Consistent with prior work, viruses and bacterial pathobionts were most prevalent in children <5 years, but children in this age group were also more likely to be tested due to symptoms of respiratory infection than for other reasons. Overall, 45.6% (213/467) of all subjects and 72% (126/175) of symptomatic subjects were <5 years of age (Table S6). To visualize the relationship between age, symptoms, and detection of virus and/or pathobiont, we plotted the number of samples from subjects with and without symptoms of respiratory infection testing positive for a virus, a pathobiont, both, or neither by age group (Fig. 2, G and H). In June/July 2021, 44.3% (78/176) of all samples were PCR-positive for viruses, pathobionts, or both; among children <5 years old, this proportion increased to 66.2% (51/77) (Table S6). Similarly, in January 2022, 53.3% (155/291) samples overall tested positive for viruses, pathobionts, or both, and within the <5 years age group, this proportion was 78.7% (107/136) (Table S6). While there were more symptomatic subjects in the <5 years age group, the enrichment in virus and pathobiont detection was also apparent in children without symptoms of acute respiratory infection. In June/July 2021, 22% (24/109) of samples from all asymptomatic children tested positive for viruses, pathobionts, or both overall; among children <5 years, the proportion was higher at 32.3% (10/31) (Fig. 2 G and Table S6). This trend was even more striking in January 2022, with 35.5% (65/183) of samples from asymptomatic children testing positive overall but 60.7% (34/56) testing positive among asymptomatic children <5 years (Fig. 2 H and Table S6). These results demonstrate a high overall prevalence of respiratory viruses and bacterial pathobionts in the nasal mucosa of children, especially children <5 years of age, regardless of the reason for SARS-CoV-2 testing.

**Figure S1.**
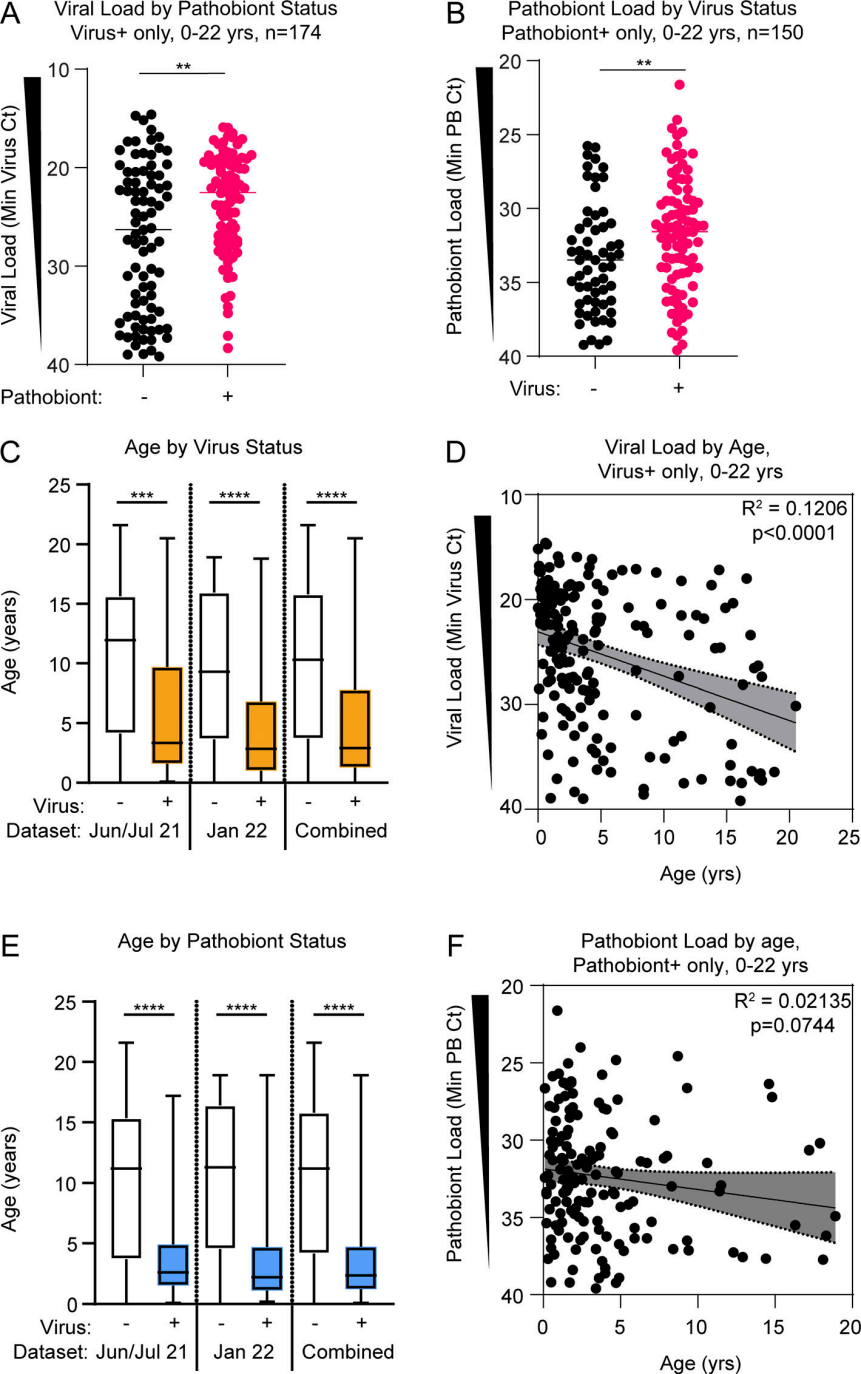
**Viral load and pathobiont load by microbe detection status.** Related to Fig. 2. **(A)** Viral load for pathobiont-negative samples (black dots, *n* = 83) and pathobiont-positive samples (magenta dots, *n* = 91) when filtered on virus-positive samples across all samples from June/July 2021 and January 2022 cohorts. Analyzed using the Mann–Whitney test. **: P = 0.0017. **(B)** Pathobiont load for virus-negative samples (black dots, *n* = 59) and virus-positive samples (magenta dots, *n* = 91) when filtered on pathobiont-positive samples across all samples from June/July 2021 and January 2022 cohorts. Analyzed using the Mann–Whitney test. **: P = 0.0098. **(C)** Subject age based on viral status for June/July 2021 (*n* = 176), January 2022 (*n* = 291), and combined cohorts (*n* = 467). Analyzed using the Kruskal–Wallis test with Dunn’s multiple comparisons tests, ***: P = 0.0001; ****: P < 0.0001. **(D)** Viral load (minimum Ct value) versus age in virus-positive samples from June/July 2021 and January 2022 cohorts (*n* = 174). R-squared goodness-of-fit and P value are shown for linear regression analysis. **(E)** Subject age based on pathobiont status for June/July 2021 (*n* = 176), January 2022 (*n* = 291), and combined datasets (*n* = 467). Analyzed using the Kruskal–Wallis test with Dunn’s multiple comparisons tests, ****: P < 0.0001. **(F)** Pathobiont load (minimum Ct value) versus age in pathobiont-positive samples from June/July 2021 and January 2022 cohorts (*n* = 150). R-squared goodness-of-fit and P value shown for linear regression analysis.

Previous work indicates that nasal bacterial pathobionts can promote susceptibility to respiratory viruses, and conversely, respiratory viruses can promote outgrowth of nasal bacterial pathobionts (Bosch et al., 2013, 2017). Therefore, we next explored whether the presence of a bacterial pathobiont correlated with higher viral load among virus-positive samples and, conversely, whether the presence of a respiratory virus correlated with higher pathobiont load among pathobiont positive samples based on PCR cycle threshold (Ct) values which inversely correlate with viral load. Among all samples from subjects aged 0–22 years, we observed a significantly higher viral load in pathobiont-positive samples and a significantly higher pathobiont load in virus-positive samples (Fig. S1, A and B). However, both pathobiont and viral prevalence and load were higher in children <5 years old (Fig. S1, C–F). Therefore, we considered the possible confounding effect of the association of young age (<5 years) on both variables. To address this, we focused exclusively on children <5 years of age. Within this age group, among virus-positive samples, pathobiont codetection did not correlate with a significant difference in viral load (Fig. 2 I), suggesting that higher viral load and pathobiont detection are associated in the larger dataset because they are both features of young age (<5 years). When we switched the focus of the age-restricted analysis to the pathobiont load, we found that the pathobiont load was still significantly higher in virus-positive samples, suggesting that viral infection is associated with an increase in the load of nasal bacterial pathobionts (Fig. 2 J).

In sum, comprehensive testing for respiratory pathogens by RT-qPCR demonstrated that in both June/July 2021 and January 2022, about one-third of children undergoing SARS-CoV-2 screening tested positive for a virus on a comprehensive respiratory virus PCR panel and about one-quarter to one-third of children tested positive for bacterial pathobionts by PCR. Most of these infections were not diagnosed during the patient care encounter but were revealed by comprehensive PCR testing agnostic of clinical presentation (Fig. 2, A–F).

### Respiratory viruses drive heightened nasal interferon responses in children

**Figure 3.**
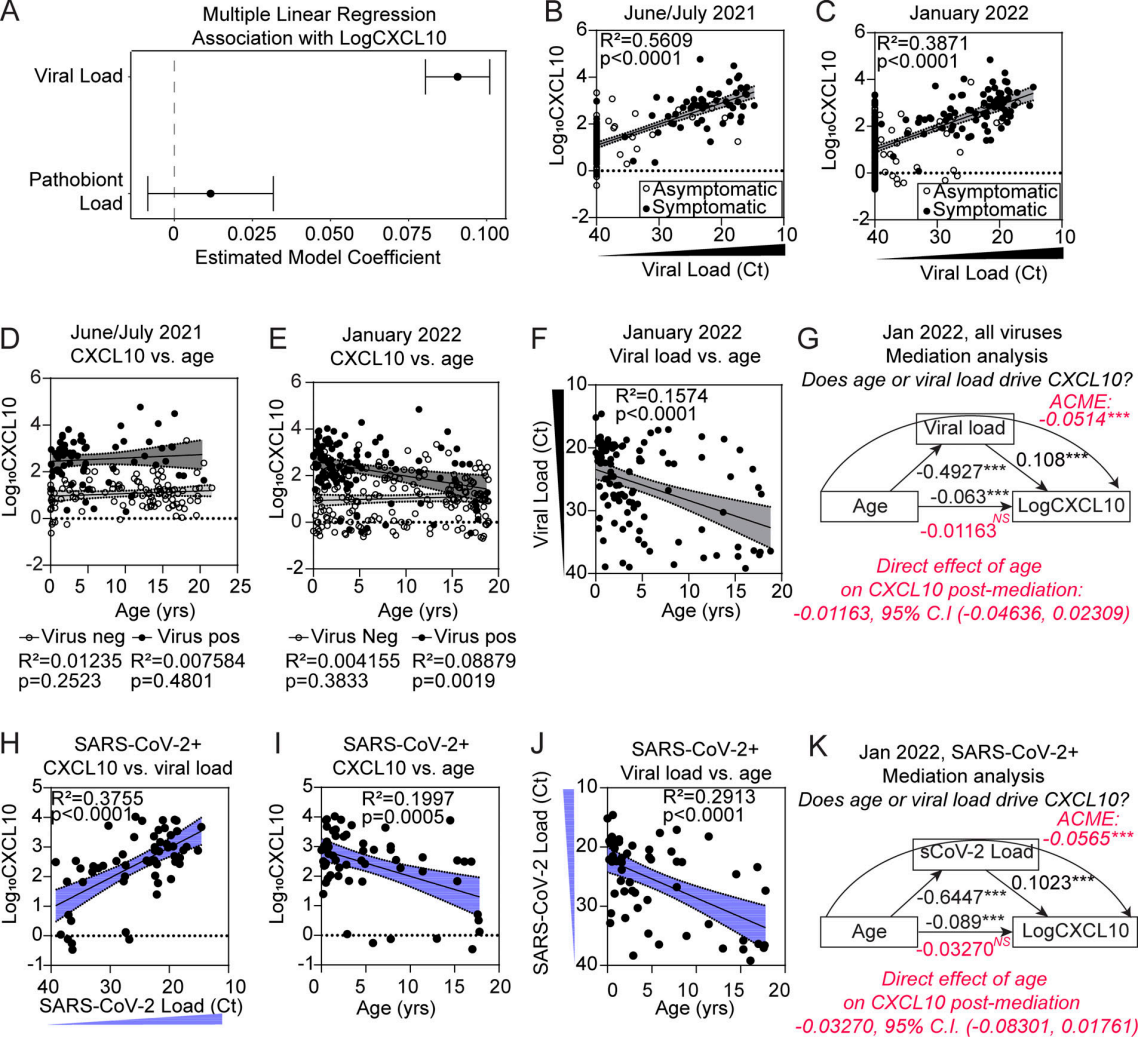
**Relationship between viral load, age, and NP CXCL10 level in pediatric subjects from June/July 2021 and January 2022. (A)** Results from multiple linear regression analysis predicting Log_10_CXCL10 (pg/ml) based on viral load and pathobiont load. 95% confidence intervals (CI) show the estimated model coefficient associated with each IV. Results are shown for combined June/July 2021 and January 2022 datasets (*n* = 467). **(B and C)** Linear regression results between Log_10_CXCL10 (pg/ml) (DV) and viral load (Ct) for June/July 2021 (*n* = 176) (B) and January 2022 (*n* = 291) (C) for subjects with (black dots) and without (white dots) symptoms. In samples with viral coinfections, the highest viral load (minimum Ct value) is plotted. R-squared and P value shown for each linear regression result. **(D and E)** Log_10_CXCL10 (pg/ml) versus age in the June/July 2021 (*n* = 176) (D) and January 2022 (*n* = 291) (E) dataset. Linear regressions are performed separately for virus-positive samples (black dots) and virus-negative samples (white dots). **(F)** Viral load (Ct) versus age for all virus-positive samples in the January 2022 dataset (*n* = 106). Data were analyzed using linear regression. R-squared and P value are shown for linear regression. **(G)** Mediation analysis results for viral load (input into model as 40-Ct) as a mediator between age and CXCL10 in virus-positive samples (*n* = 106). Figure depicts the ACME and associated 95% CI through viral load denoted by the curved arrow (−0.0514 [−0.0831, −0.03], P < 2 × 10^–16^) and the ADE of age on CXCL10 (−0.0116 [−0.0484, 0.03], P = 0.56). 95% CIs obtained from 1,000 bootstrapped samples. **(H)** Log_10_CXCL10 (pg/ml) versus viral load (Ct) in SARS-CoV-2–positive samples (single viral infection only, *n* = 57). Data were analyzed using linear regression with the 95% CI shown. R-squared and P values are shown for linear regression. **(I)** Log_10_CXCL10 (pg/ml) versus age in SARS-CoV-2–positive samples (*n* = 57). Data are analyzed using linear regression with the 95% CI shown. R-squared and P value are shown for linear regression. **(J)** SARS-CoV-2 viral load versus age in SARS-CoV-2–positive samples (*n* = 57). Data were analyzed using linear regression with the 95% CI shown. R-squared and P value are shown for linear regression. (**K**) Mediation analysis results for SARS-CoV-2 load (Ct value) as a mediator between age and CXCL10 in SARS-CoV-2–positive samples (*n* = 57). Figure depicts the ACME and associated 95% CI through viral load denoted by the curved arrow (−0.0500 [−0.0972, −0.03], P < 2 × 10^–16^) and the ADE of age on CXCL10 (−0.0106 [−0.0886, 0.03], P = 0.274). 95% CIs obtained from 1,000 bootstrapped samples.

Next, we sought to define the relationship between respiratory pathogen detection and activation of nasal innate immune responses in children by comparing pathogen RT-qPCR results to biomarkers indicative of innate immune activation in the same samples. Previous work from our group showed that the interferon-inducible protein CXCL10 is a robust biomarker of the nasal interferon response and that CXCL10 levels in the NP swab–associated viral transport media directly correlate with ISG mRNA expression by RNA-seq (Cheemarla et al., 2021; Landry and Foxman, 2018). Therefore, to estimate activation of the mucosal interferon response in the upper respiratory tract, we measured NP swab–associated CXCL10 protein in all samples using a clinical-grade microfluidics-based immunoassay (Aldo et al., 2016; Cheemarla et al., 2023a). Consistent with our previous observations in adults, multiple linear regression analysis showed that log_10_-transformed concentration of nasal CXCL10 significantly correlated with viral load but not pathobiont load, indicating that viral presence and load primarily drive the nasal interferon response (Fig. 3 A). Biological sex did not influence nasal CXCL10 (Mendeley data [Watkins and Foxman, 2024]). Nasal CXCL10 correlated with viral load similarly in both collection periods despite differences in circulating viruses (Fig. 2, C and D; and Fig. 3, B and C). Viral load and nasal CXCL10 tended to be lower in children without symptoms of respiratory infection compared with symptomatic subjects (white vs. black circles, Fig. 3, B and C, and Mendeley data [Watkins and Foxman, 2024]). These data indicate a wide range of activity of the nasal mucosal interferon response in children and show that, within this age group, the activity of this response correlates with the presence and viral load of a respiratory virus.

### Viral load, not age, is the main driver of heightened nasal interferon response in children positive for SARS-CoV-2 or other respiratory viruses

Next, we sought to define the relationship between nasal respiratory viruses and the nasal interferon response using CXCL10 as a biomarker. In June/July 2021, when RV was the predominant virus detected, CXCL10 was elevated in virus-positive compared with virus-negative samples, but CXCL10 did not correlate with age within either group (Fig. 3 D). Together with the strong correlation of nasal CXCL10 to viral load (Fig. 3 B), these data point to virus presence and load, not age, as the main driver of the nasal interferon response in these samples.

Next, we examined samples from the January 2022 Omicron surge in which the predominant virus detected was SARS-CoV-2. There was no relationship between nasal CXCL10 and age in virus-negative subjects (white circles, Fig. 3 E). However, among virus-positive subjects, CXCL10 concentration was slightly but significantly higher in younger children (black circles, Fig. 3 E). We also observed a correlation between younger age with higher viral load in January 2022 (Fig. 3 F), which could be due to a number of factors including a lack of approved vaccines for SARS-CoV-2 for children under the age of 5 years at that time in our region. In contrast, vaccines were available for older children, with an estimated rate of vaccine coverage in Connecticut of 36.8% partially or fully vaccinated for ages 5–11, 75.4% for ages 12–15, and 79.3% for ages 16–24 (Connecticut Department of Public Health, 2023).

To determine which variable, viral load or age, was the stronger driver of nasal CXCL10 level in January 2022, we applied mediation analysis with multiple regression (Baron and Kenny, 1986), which showed that viral load fully mediated the effect of age on CXCL10 level (average causal mediation effect [ACME] with p < 2 × 10^−16^, curved arrow, Fig. 3 G). We also examined the relationship between viral load, nasal CXCL10, and age for SARS-CoV-2^+^ samples only, which also showed significant positive correlations between viral load and CXCL10, young age and CXCL10, and young age and viral load (Fig. 3, H–J). Mediation analysis on this subset also showed that viral load fully mediated the effect of age on CXCL10 (significant ACME with p < 2 × 10^−16^, curved arrow, Fig. 3 K). Bootstrapping analysis confirmed these results showed a nonsignificant average direct effect (ADE) of age on CXCL10 for virus-positive samples (Fig. 3, G and K, red text). These results indicate that the higher nasal CXCL10 levels observed in younger virus-positive children during January 2022 are due to a confounding effect of higher viral loads in younger children rather than a direct intrinsic effect of young age. Overall, these data show that viral presence and load, rather than subject age, predict the activity of the nasal mucosal interferon response in children.

### Coinfection with another respiratory virus in SARS-CoV-2^+^ subjects is associated with a more robust mucosal antiviral response than predicted by SARS-CoV-2 viral load alone

Previous work using in vitro models of the differentiated respiratory epithelium has shown that coinfection with another respiratory virus can enhance the interferon response in virus target cells during SARS-CoV-2 infection, but it is unclear whether this happens in human subjects (Cheemarla et al., 2021, 2023b, Dee et al., 2021, 2023; Essaidi-Laziosi et al., 2022; Fage et al., 2022). In the June/July 2021 and January 2022 datasets, there were a total of 17 viral coinfections. To increase the sample size for assessing the effect of viral coinfections on the mucosal innate immune response, we performed comprehensive virology testing and CXCL10 immunoassay on an additional 167 residual NP samples from symptomatic children undergoing testing for SARS-CoV-2 or other viruses in August 2021 during respiratory syncytial virus (RSV) resurgence in our region. The combined set of 634 samples (June/July 2021, August 2021, January 2022) included 42 viral coinfections. Using these samples, we compared nasal CXCL10 in single infections and coinfections with the two most prevalent viruses in the combined sample set: SARS-CoV-2 and RV.

**Figure 4.**
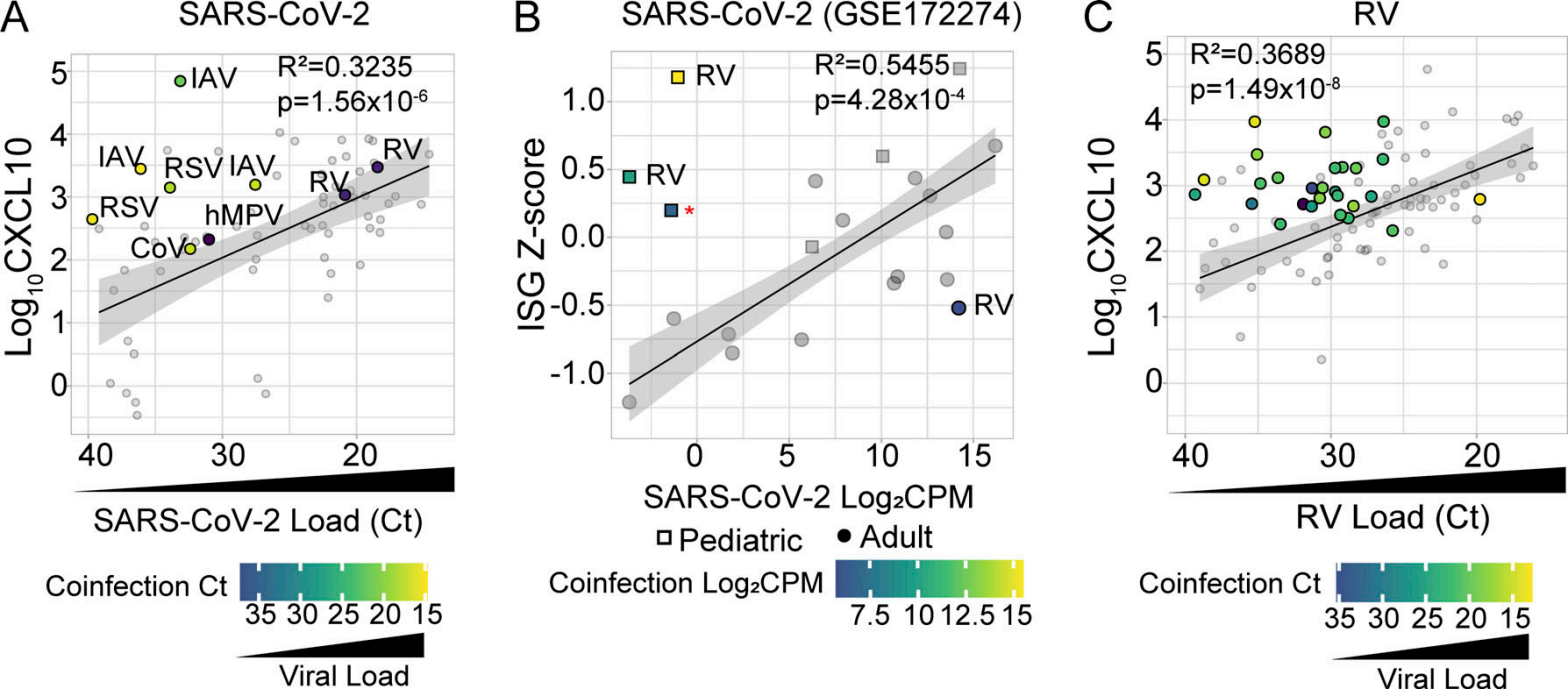
**NP innate immune response in SARS-CoV-2**^**+**^ **and RV**^**+**^ **subjects with and without co-infecting respiratory viruses. (A)** NP Log_10_CXCL10 (pg/ml) versus SARS-CoV-2 viral load (Ct value) in samples with viral coinfections (colored dots, *n* = 9) and without viral coinfections (gray dots, *n* = 59) from June/July 2021, August 2021, and January 2022 cohorts. Linear regression is performed on SARS-CoV-2 single infections only with the 95% CI shown. R-squared and P value of linear regression analysis are added to the top right corner. Dots in viral coinfections are color-coded according to the viral load of the coinfecting virus. IAV = influenza A virus; CoV = seasonal coronavirus; hMPV = human metapneumovirus. **(B)** Viral load (Log_2_-CPM) versus nasal RNA-seq ISG Z-score for samples with and without SARS-CoV-2 viral coinfections in the GSE172274 dataset. Linear regression is performed on SARS-CoV-2 single infections only (gray symbols) with the 95% CI shown. R-squared and P value of linear regression analysis are added to the top right corner. Children are represented as squares while adults are represented as dots. Red asterisk = measles virus (Moraten vaccine strain). **(C)** NP CXCL10 (Log_10_ pg/ml) versus RV viral load (Ct value) in samples with viral coinfections (colored dots, *n* = 27) and without viral coinfections (gray dots, *n* = 70) from June/July 2021, January 2022, August 2021, and January 2022 cohorts. Linear regression is performed on RV single infections only (gray symbols) with the 95% CI shown. R-squared and P value of linear regression analysis are shown. Symbols in viral coinfections are color-coded according to the viral load of the coinfecting virus.

First, we examined viral coinfections with SARS-CoV-2. There were 68 SARS-CoV-2^+^ samples, and 9 had a coinfecting respiratory virus. We compared nasal CXCL10 to viral load in samples with SARS-CoV-2 single infections (Fig. 4 A, gray dots) to SARS-CoV-2^+^ samples with viral coinfections (Fig. 4 A, colored dots). For all samples with a coinfecting respiratory virus, CXCL10 values were above the regression line between SARS-CoV-2 viral load and CXCL10 for single infections indicating a more robust activation of the mucosal interferon response in coinfections (Fig. 4 A). Samples with high viral loads of coinfecting viruses (e.g., IAV and RSV coinfections) and low SARS-CoV-2 loads had CXCL10 values far above the regression line predicted by single infections. CXCL10 fell much closer to values expected for SARS-CoV-2 single infections when subjects had high viral loads of SARS-CoV-2 and low viral loads of coinfecting viruses, consistent with the nasal interferon response being predominantly driven by the virus with higher viral load.

**Figure S2.**
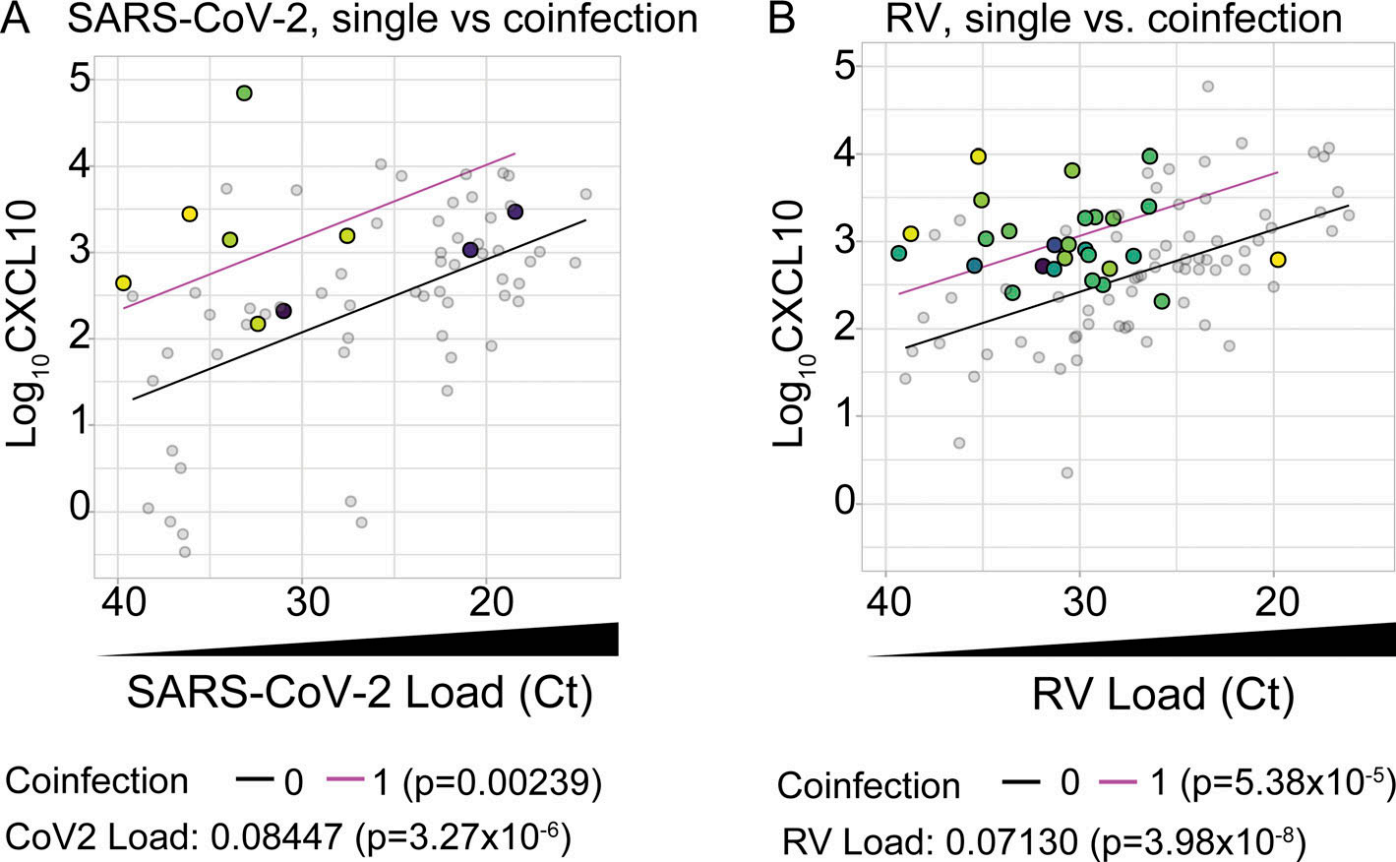
**Multiple linear regression model results of CXCL10 versus viral load in single- and co-infections.** Related to Fig. 4. **(A and B)** Multiple linear regression analysis with viral coinfections as a binary predictor variable (0 = no viral coinfection; 1 = viral coinfection) and viral load (input as 40-Ct) as a continuous predictor variable using data shown in Fig. 4, A and C. Model results are shown as separate lines for SARS-CoV-2 (A) or RV (B) single infections (black line) and viral coinfections (magenta). P-values for each predictor variable in multiple linear regression analysis are shown below the graphs.

To statistically test whether viral coinfections increased the CXCL10 response, we used multiple linear regression analysis to predict CXCL10 in single infections vs. coinfections, using SARS-CoV-2 load (40-Ct) as a continuous predictor variable and the presence of any viral coinfection as a binary predictor variable (0 = no viral coinfection; 1 = viral coinfection) (Fig. S2 A). Consistent with the results shown in Fig. 3, increasing SARS-CoV-2 load was significantly associated with an increase in CXCL10 protein. Additionally, the presence of a coinfecting virus was independently associated with higher CXCL10 protein compared with single infections (Fig. S2 A). Fig. 4 A also shows several examples of cases in which children were diagnosed with COVID-19 based on a positive SARS-CoV-2 PCR test, but in fact comprehensive testing showed they had low viral loads of SARS-CoV-2 and much higher loads of other viruses.

Prior published reports of heightened nasal innate immunity in SARS-CoV-2^+^ children showed no correlation between the nasal interferon response and SARS-CoV-2 viral load, but the presence of other viruses was not comprehensively evaluated (Loske et al., 2022; Pierce et al., 2021; Wimmers et al., 2023; Winkley et al., 2021; Yoshida et al., 2022). To further probe whether coinfections play a role in the discordance between nasal interferon responses and SARS-CoV-2 viral load in children, we analyzed RNA-seq data from a previous study showing heightened nasal innate immunity in SARS-CoV-2^+^ children (GSE172274). We used an ISG mRNA expression signature to estimate the nasal interferon response and metagenomics to identify viral reads as previously described (Cheemarla et al., 2023a; Cunningham et al., 2022; Kalantar et al., 2020; Langmead and Salzberg, 2012; Pierce et al., 2021) (Fig. 4 B). Coinfecting viruses were identified in three out of six children, all <5 years old, and included two subjects with high RV viral loads (14,575 reads per million [rPM] and 1,036 rPM) and SARS-CoV-2 reads near or below the limit of detection for RNA-seq (0.4902 rPM and 0 rPM) (Fig. 4 B, squares). One sample contained reads from the vaccine strain of measles virus consistent with the age of the subject being typical for the first measles, mumps, rubella dose and case reports that vaccine measles virus can temporarily be found in the nasopharynx after vaccination (Kaic et al., 2010; Kobune et al., 1995; Morfin et al., 2002). In contrast, only one viral coinfection was identified among the 15 adults with nasal RNA-seq data, with a low RV viral load (52 rPM) and a high viral load of SARS-CoV-2 (18,821.5 rPM) (Fig. 4 B, dots). ISG Z-score correlated with SARS-CoV-2 viral load in subjects with SARS-CoV-2 and no other virus detected; however, the three children with viral coinfections had higher ISG Z-scores than expected based on regression analysis of single infections (Fig. 4 B). While the number of subjects is limited, these findings support the conclusion that coinfecting viruses drive an augmented mucosal interferon response during SARS-CoV-2 infection and that more frequent viral coinfections in children may contribute to the previously described heightened nasal interferon responses during SARS-CoV-2 infection.

We also evaluated whether coinfection enhanced the nasal interferon response in subjects with RV infection. In the combined dataset, we identified a total of 97 samples positive for RV, including 27 coinfections: 25 of these samples had one additional virus detected and two samples had two additional viruses detected. RV coinfections included diverse viruses such as RSV (*n* = 16), parainfluenza (*n* = 9), adenovirus (*n* = 2), and SARS-CoV-2 (*n* = 2). Similar to what we observed for SARS-CoV-2, samples with a viral coinfection had higher nasal CXCL10 than predicted by RV infection alone, especially when the coinfecting virus was present at a high viral load (Fig. 4 C). Multiple linear regression analysis using RV load as a continuous predictor variable, viral coinfection as a binary predictor variable (0 = no viral coinfection; 1 = viral coinfection), and CXCL10 as the response variable showed that RV load was significantly correlated with CXCL10 and that viral coinfection drove a significant increase in CXCL10 protein measured compared with single infections (Fig. S2 B). Together, these results reinforce the conclusion that viral load in single infections, or the combined viral load in coinfections, drives the interferon response in the nasal mucosa, and that discordance between SARS-CoV-2 viral load and nasal interferon response noted in prior reports may have been due to undiagnosed coinfecting viruses.

### Bacterial pathobionts alter the nasal mucosal immunophenotype

**Figure 5.**
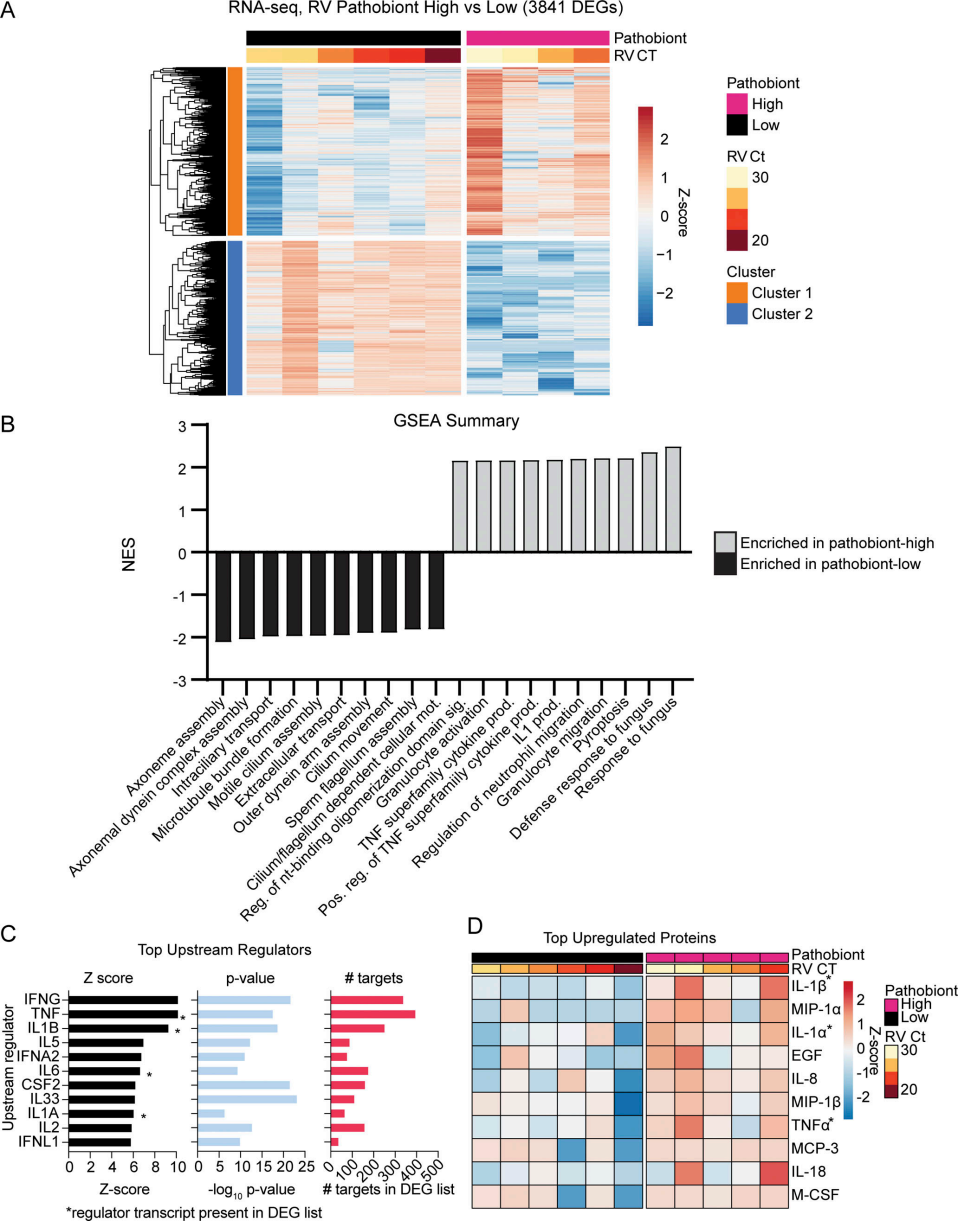
**RNA-seq analysis of RV-positive pathobiont-high vs. pathobiont-low NP swab samples. (A)** DEGs between RV-positive pathobiont-high (*n* = 4) and RV-positive pathobiont-low (*n* = 6) samples from the dbGaP RNA-seq dataset phs002442.v1.p1. Genes enriched in pathobiont-high samples are shown in cluster 1 (orange); genes enriched in pathobiont-low samples are shown in cluster 2 (blue). DEGs are filtered by |Log_2_FC| >1, FDR <0.05. Pathobiont-high and pathobiont-low samples are defined at >10^5^ rPM and <10^4^ rPM respectively of *M. catarrhalis*, *H*. *influenzae*, or *S. pneumoniae*. **(B)** GSEA results comparing RV-positive pathobiont-high to RV-positive pathobiont-low samples. The top 10 pathways enriched for each pathobiont level are shown and plotted according to the normalized enrichment score (NES). RV-positive pathobiont-high enriched pathways are gray; RV-positive pathobiont-low enriched pathways are black. **(C)** Ingenuity pathway analysis (IPA) prediction of upstream regulators of DEGs found in A. Upstream regulators are filtered on cytokines and listed in order of descending Z-score. Asterisks denote cytokines which were also upregulated, differentially expressed transcripts. **(D)** Top differentially expressed, upregulated cytokines in RV-positive pathobiont-high samples (*n* = 5) compared with RV-positive pathobiont-low samples (*n* = 6). Top cytokines are identified using two-group *t* test comparison and are listed as the top 10 proteins in order of most significant q-value. Asterisks denote cytokines identified as upstream regulators in C.

We next sought to explore whether bacterial pathobiont detection correlates with changes in nasal mucosal innate immune activation. To select NP cytokine biomarkers that reflect pathobiont-induced mucosal innate immune responses, we reexamined a previous set of NP samples from RV-infected children and adults (Cheemarla et al., 2023a). About half of these samples had high loads of bacterial pathobionts based on RNA-seq data. Analysis of differentially expressed genes (DEGs) between RV-positive pathobiont-low and RV-positive pathobiont-high samples identified 3,841 significant DEGs (|log_2_FC| >1, false discovery rate [FDR] <0.05) (Fig. 5 A). Gene set enrichment analysis (GSEA) showed that pathways upregulated in RV-positive pathobiont-high samples included neutrophil/granulocyte activation and migration, immune response to bacterial and fungal pathogens, pyroptosis, and IL-1 and TNF family cytokine production (Fig. 5 B). Pathways enriched in RV-positive, pathobiont-low samples included cilium assembly and movement, suggesting relative enrichment for ciliated respiratory epithelial cells. Ingenuity pathway analysis was used to predict upstream cytokine regulators of the RV-positive pathobiont-high vs. -low phenotype. Among the top 10, four were also significantly enriched at the mRNA level: TNF, IL-1β, IL-6, and IL-1α (Fig. 5 C, asterisks). Multiplex testing for 71 cytokines revealed that TNF, IL-1β, and IL-1α were also among the top 10 differentially enriched cytokines at the protein level (Fig. 5 D, asterisks). IL-1β and TNF were also shown to be associated with *M. catarrhalis* and *H. influenzae* colonization in a prior study of 662 asymptomatic infants, suggesting that bacterial pathobionts also induce these cytokines in the nasal mucosa without RV infection, although the presence of viruses was not assessed in the prior study (Folsgaard et al., 2013).

### Cytokine landscape of nasal innate immune activity in children <5 years old shows distinct patterns related to viral and bacterial pathobiont burden

To evaluate nasal TNF and IL-1β proteins as biomarkers of the nasal mucosal innate immune response to bacterial pathobionts and their relationship to virus-induced interferon responses, we tested associations between microbial drivers and NP protein levels of CXCL10, IL-1β, and TNF. We focused on children <5 years in whom pathobionts were most prevalent. We compared four groups of subjects in the combined June/July 2021 and January 2022 datasets: virus-negative, pathobiont-negative (*n* = 55); virus-positive, pathobiont-negative (*n* = 40); virus-negative, pathobiont-positive (*n* = 38); and virus-positive, pathobiont-positive (*n* = 79).

**Figure 6.**
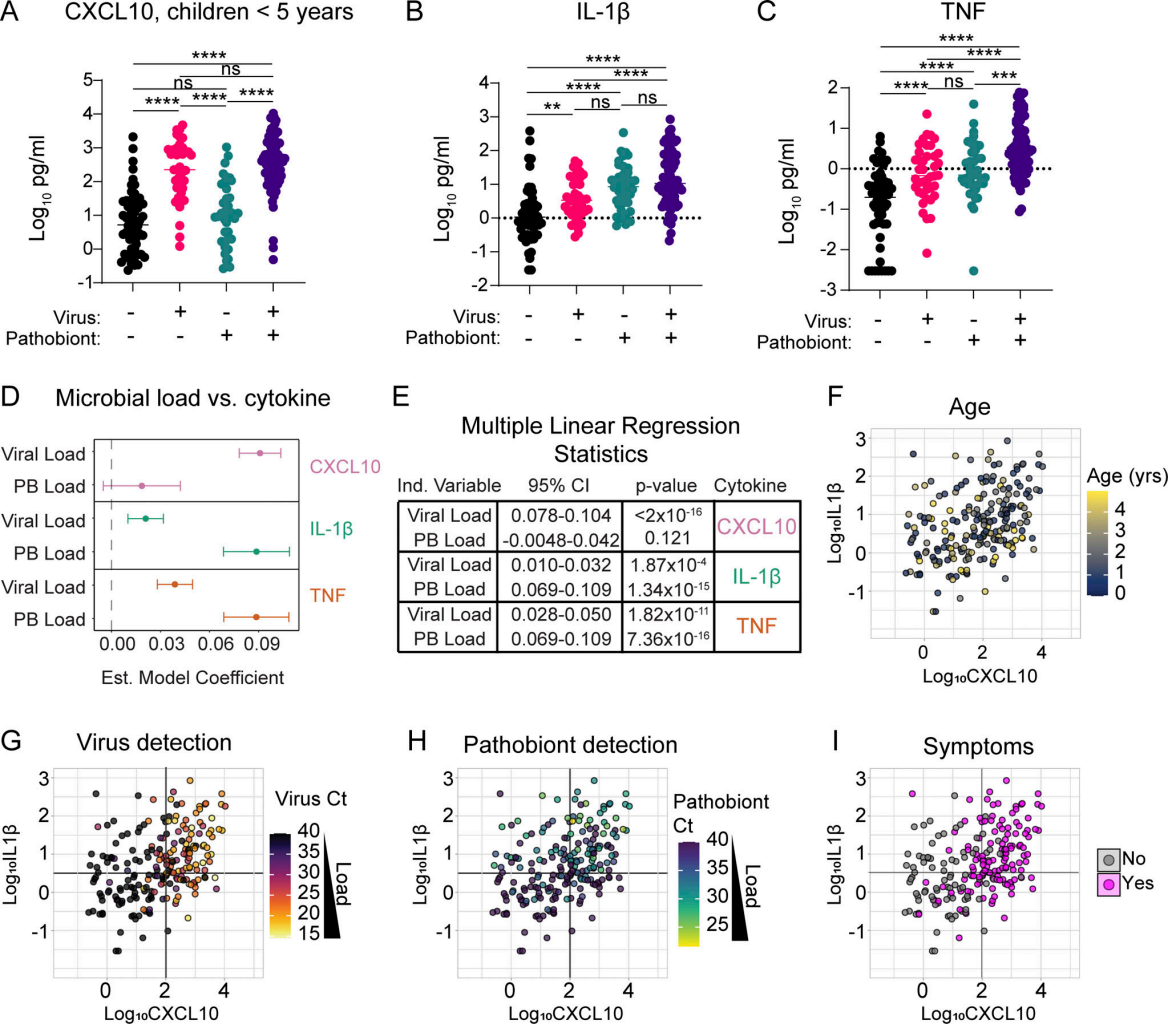
**Immunophenotyping of nasal samples from children <5 years old. (A–C)** Levels of NP CXCL10 (Log_10_ pg/ml), IL-1β (Log_10_ pg/ml), and TNF (Log_10_ pg/ml) among viral and pathobiont categorical groups in children <5 years from June/July 2021 and January 2022 cohorts (*n* = 212). Data were analyzed using Welch’s ANOVA test with Dunnett’s T3 multiple comparisons tests. ns = not significant (P > 0.05), ** = P < 0.01 *** = P < 0.001, **** = P < 0.0001. **(D and E)** Forest plot (D) and multiple linear regression analysis results (E) showing estimated model coefficients between cytokines and viral or pathobiont load in children <5 years (*n* = 212). Each cytokine is analyzed as a response variable, and pathobiont load and viral load are input as predictor variables. 95% CI and P values are shown for each estimated model coefficient. **(F–I)** Levels of NP CXCL10 (Log_10_ pg/ml) versus IL-1β (Log_10_ pg/ml) in subjects <5 years of age (*n* = 212). Dot color depicts age (F), viral Ct (G), pathobiont Ct (H), or the presence of respiratory symptoms (I).

Consistent with our observations in subjects aged 0–22 years (Fig. 3 A), in children <5 years, we observed a significant elevation of nasal CXCL10 in virus-positive samples independent of pathobiont status, and pathobiont detection alone was not associated with CXCL10 elevation (Fig. 6 A). These data reaffirm that a robust mucosal interferon response, as indicated by NP CXCL10, is triggered by viral infection. In contrast, IL-1β showed a slight but significant increase in virus-only detection but was significantly more elevated in pathobiont-only detection or virus/pathobiont codetection (Fig. 6 B). TNF was significantly elevated in virus-only or pathobiont-only samples compared to negative controls and showed further elevation in virus/pathobiont codetection compared with pathobiont-only samples (Fig. 6 C). These results indicate that IL-1β and TNF can be induced by either viruses or bacterial pathobionts, but they are more strongly associated with the mucosal response to bacterial pathobionts.

**Figure S3.**
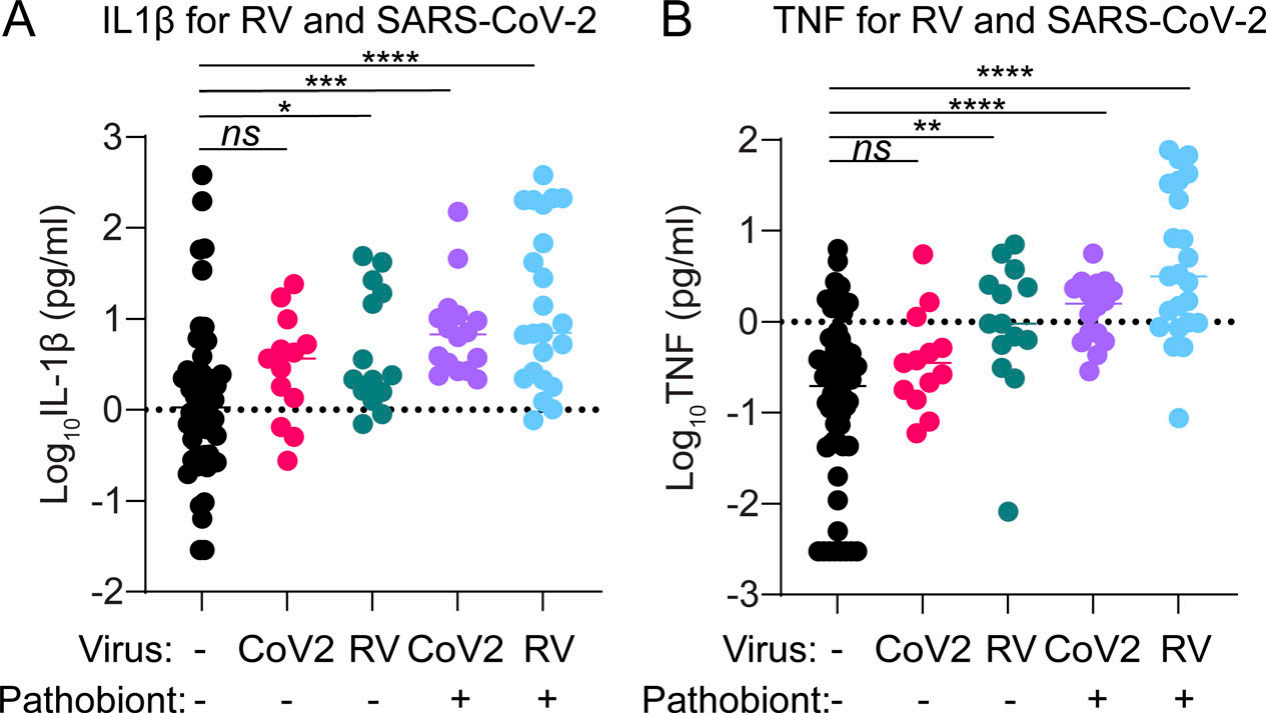
**IL-1β and TNF for RV and SARS-CoV-2 in children <5 years.** Related to Fig. 6. **(A)** Log_10_ IL-1β (pg/ml) is shown for virus/pathobiont-negative samples (*n* = 55), RV^+^ samples without pathobionts (*n* = 13), SARS-CoV-2^+^ samples without pathobionts (*n* = 14), RV^+^ samples with pathobionts (*n* = 16), and SARS-CoV-2^+^ samples with pathobionts (*n* = 23). Data were analyzed using Welch’s ANOVA with Dunnett’s T3 multiple comparisons. ns = not significant (P > 0.05); * = P < 0.05; *** = P < 0.001; **** = P < 0.0001. **(B)** Log_10_ TNF (pg/ml) is shown for virus/pathobiont-negative samples (*n* = 55), RV^+^ samples without pathobionts (*n* = 13), SARS-CoV-2^+^ samples without pathobionts (*n* = 14), RV^+^ samples with pathobionts (*n* = 16), and SARS-CoV-2^+^ samples with pathobionts (*n* = 23). Data were analyzed using Welch’s ANOVA with Dunnett’s T3 multiple comparisons. ns = not significant (P > 0.05); * = P < 0.05; ** = P < 0.01; **** = P < 0.0001.

Since different viruses can induce different patterns of innate immune activation, we also performed this analysis individually for the two viruses most prevalent in our datasets: SARS-CoV-2 and RV (Fig. S3, A and B). Both SARS-CoV-2 and RV infection alone led to a slight increase in levels of IL-1β and TNF, but this trend was statistically significant only for RV. However, both SARS-CoV-2 and RV infections showed a clear and significant enhancement of IL-1β and TNF during virus–pathobiont codetection. These data suggest that while SARS-CoV-2 and RV infections weakly induce pathways connected to IL-1β and TNF alone, the presence of pathobionts promotes production of these cytokines, and virus–pathobiont codetection with either virus enhances these responses.

Since categorical analysis does not account for the influence of viral or pathobiont load on nasal cytokine concentration, we used multiple linear regression to estimate the relationship between viral load and pathobiont load as predictor variables and each cytokine as the response variable. Nasal CXCL10 is significantly associated with viral load and not with pathobiont load. Conversely, NP IL-1β and TNF were statistically significantly associated with both pathobiont load and viral load consistent with our categorical analysis (Fig. 6, D and E).

**Figure S4.**
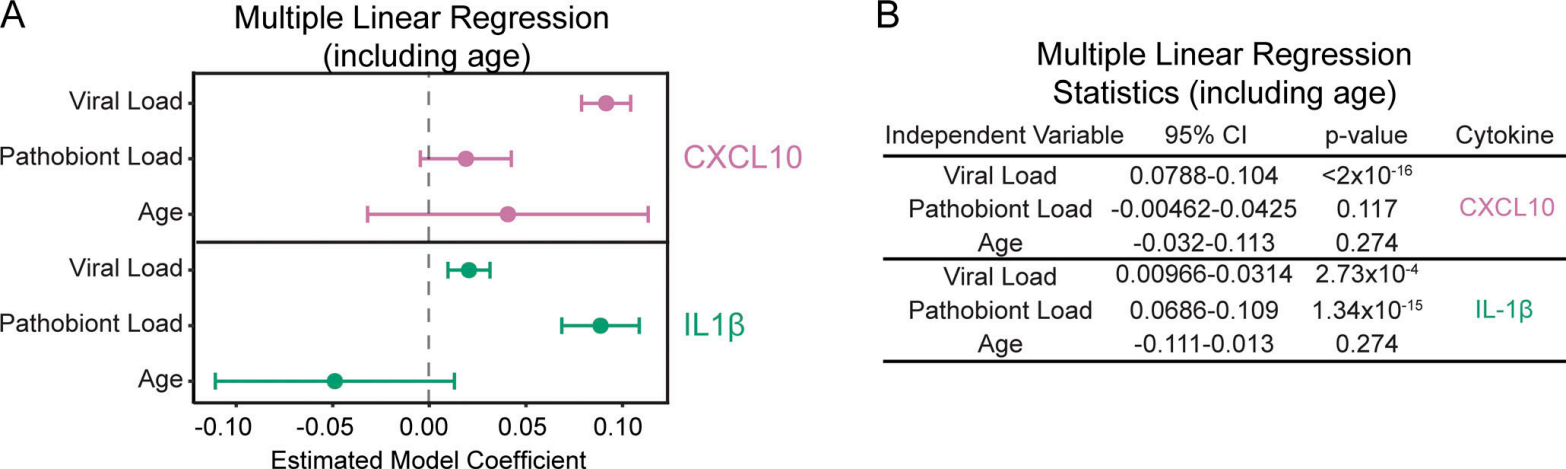
**Multiple linear regression results for CXCL10 and IL-1β in children <5 years (*n* = 212) using viral load, pathobiont load, and age as predictors.** Related to Fig. 6. **(A and B)** The results of the model are summarized using a forest plot (A) and table (B). TNF is not shown due to poor goodness-of-fit testing of the model.

To visualize the association between nasal innate immune activation and age, viral load, pathobiont load, and symptoms in children <5 years, we generated scatterplots of samples based on nasal CXCL10 vs. IL-1β. Within this subset, age did not correlate with cytokine pattern when accounting for viral and pathobiont load (Fig. 6 F and Fig. S4). In contrast, plots highlight the strong association between viral load and nasal CXCL10 and between pathobiont load and IL-1β (Fig. 6, G and H). Children with respiratory symptoms were separated into a CXCL10-high and IL-1β low-to-high phenotype, consistent with viral load being the variable most associated with respiratory symptoms (Fig. 6 I).

### Nasal immunophenotype changes dynamically with viral load in paired longitudinal samples from healthy 1-year-olds

**Figure 7.**
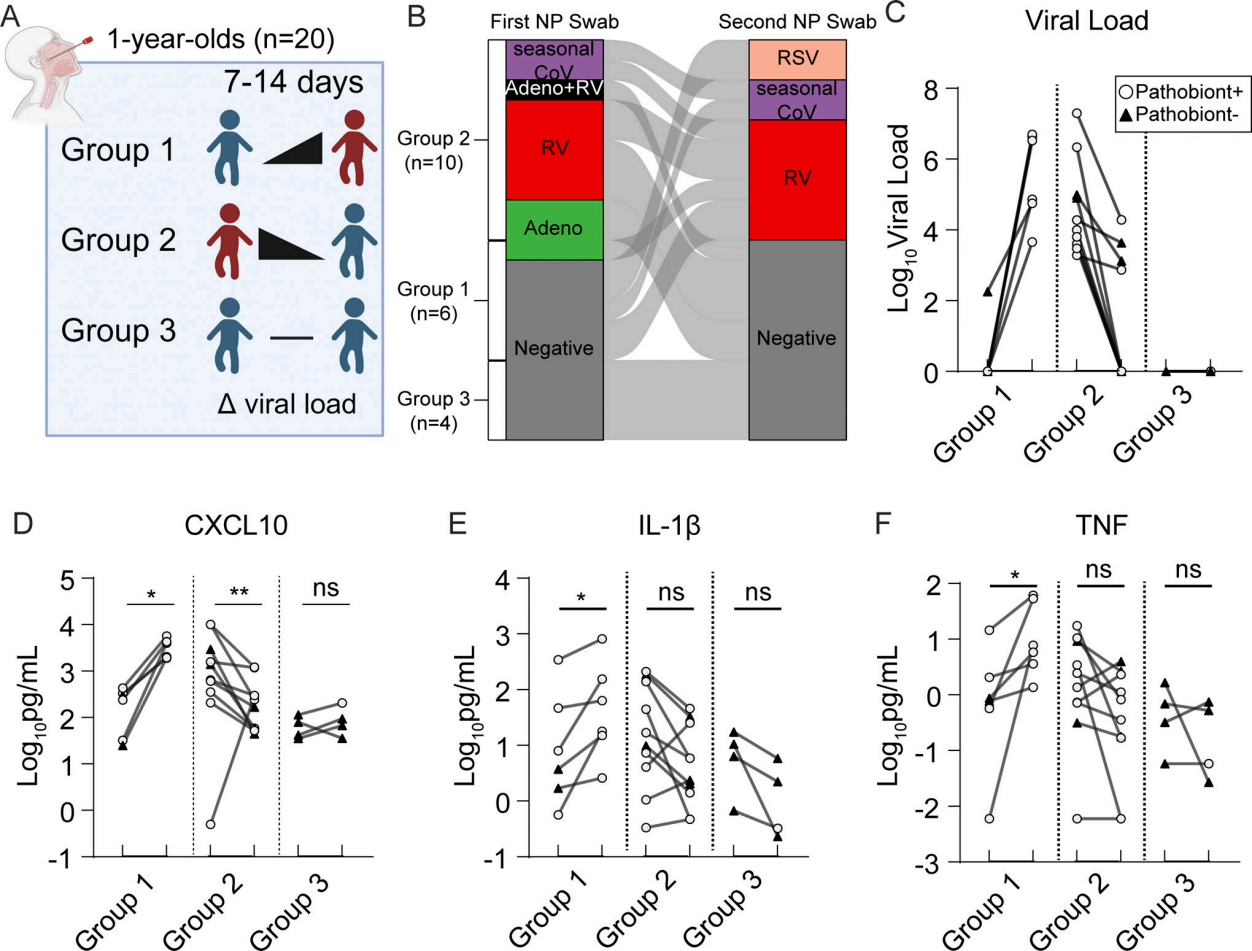
**Analysis of paired NP swabs from 1-year-old children presenting for well visits. (A)** NP swabs were collected from 1-year-old children (*n* = 20) presenting to the pediatric clinic for well visits. One NP swab was collected at the time of the appointment, and a second NP swab was collected 7–14 days later from the same children. Children were separated into three groups based on respiratory viral panel results: Group 1 includes children who experienced the acquisition of or increase in viral load between the first and second visits (*n* = 6); Group 2 includes children who experienced a decrease in viral load between the first and second visits (*n* = 10); Group 3 includes children who were virus-negative on both visits (*n* = 4). **(B)** Dynamics of respiratory viral panel results are shown as an alluvial diagram for each group of 1-year-old children for NP swabs taken on the first and second visits. **(C)** Log_10 _viral load (based on Ct value relative to detection limit) is shown for each group of children. **(D–F)** Cytokine protein concentrations are plotted for each group as a Log_10_ pg/ml transformed values for CXCL10 (D), IL-1β (E), and TNF (F). Paired samples are analyzed using the Wilcoxon signed-rank test on non-scaled cytokine concentrations. ns: not significant (P > 0.05); *: P = 0.0312; **: P = 0.0059.

**Figure S5.**
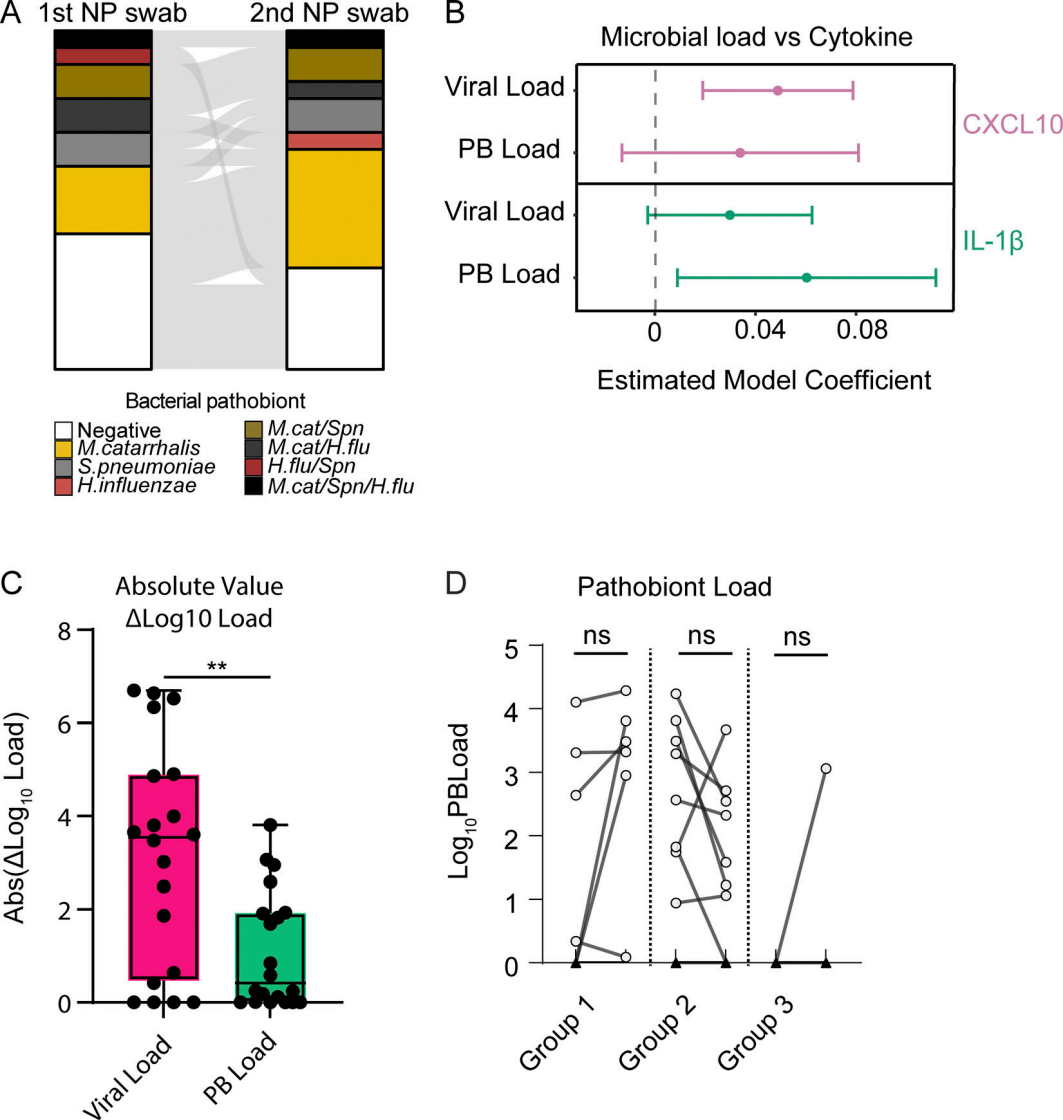
**Virus and pathobiont test statistics and changes in load across sample pairs. (A)** Alluvial diagram showing pathobiont test results for first and second NP swabs (*n* = 20 pairs). **(B)** Multiple linear regression analysis results on the paired-sample dataset (*n* = 40 samples). Multiple linear regression models were separately performed for CXCL10 (Log_10_ pg/ml) and IL-1β (Log_10_ pg/ml) based on viral load (40-Ct) and pathobiont load (40-Ct) as predictor variables. A regression model for TNF was performed but excluded due to poor goodness-of-fit testing. For CXCL10, P values are the following: “Viral Load”: P = 0.00288; “Pathobiont Load”: P = 0.16855. For IL-1β, P values are the following: “Viral Load”: P = 0.0830; “Pathobiont Load”: P = 0.0276. **(C)** Change in microbial load between samples (Post-Pre, Log_10_-transformed absolute value) for viral load (magenta) and pathobiont load (green). The Mann–Whitney test is used to compare changes in load. *: P = 0.0091. **(D)** Pathobiont load (Log_10_-transformed, relative to detection limit) for sample pair groups 1–3. Paired samples are analyzed using the Wilcoxon signed-rank test. ns = not significant (P > 0.05).

To further test the idea that respiratory viruses drive heightened nasal innate immunity in young children, we collected paired NP samples from 20 1-year-olds presenting to the pediatrician for routine healthy child visits during September 2022–September 2023. Children were recruited to donate paired longitudinal NP samples, one at the time of the routine visit and a second swab 7–14 days later (median interval of 8 days) (Fig. 7 A). All samples underwent comprehensive virology and bacterial pathobiont testing by RT-qPCR and immunoassay for CXCL10, IL-1β, and TNF. Surprisingly, given that samples were collected at routine well-child visits or return visits as part of this study, 24/40 samples had at least one respiratory virus detected, with the predominant viruses being RV, seasonal coronaviruses, and adenovirus (Fig. 7, B and C). Only four subjects were virus-negative in both samples. Similarly, 26/40 samples were positive for bacterial pathobionts with only five children testing negative for pathobionts in both samples (Fig. S5 A). Multiple linear regression analysis of these 40 samples showed that CXCL10 was associated with viral load and IL-1β was associated with pathobiont load, validating our independent analysis of the cross-sectional 2021–22 samples from children <5 years (Fig. S5 B and Fig. 6 D).

Viral load was significantly more dynamic than pathobiont load. The median absolute change between samples was ∼3,500-fold for viral load and ∼2.6-fold for pathobiont load (Fig. S5 C). Therefore, we focused on the effect of viral dynamics on changes in nasal cytokine immunophenotype between paired samples. Samples were categorized into three groups: children with no virus/low viral load in the first sample with increased viral load in the second sample (group 1, *n* = 10), children who were virus-positive for the first sample and had decreased viral load/no virus detected on the second sample (group 2, *n* = 6), and children with no virus in either sample (group 3, *n* = 4) (Fig. 7, A–C). CXCL10 significantly increased with viral load in all group 1 samples, significantly decreased with decreasing viral load in group 2 samples collectively and individually, apart from one outlier, and showed no significant change in group 3 samples in which no viruses were detected (Fig. 7 D). These results are consistent with the viral load being closely linked to the activity of the nasal interferon response. For IL-1β, TNF, and pathobiont load, there was no significant directional change with viral dynamics in groups 2 or 3. However, IL-1β and TNF showed a significant increase with increasing viral load in group 1 consistent with cross-sectional data (Fig. 7, E and F; and Fig. S5 D) showing that viral infection is associated with an increase in these proinflammatory cytokines, particularly in the setting of pathobiont coinfection which was also found in almost all group 1 samples (white circles, Fig. 7, C–F).

These data reinforce the association between nasal viral load and mucosal interferon response seen in cross-sectional samples and show that viral load drives nasal immunophenotype even when other biological variables are fixed. Moreover, these data reveal that heightened mucosal innate immunity is not a static phenotype but rather changes in parallel with the acquisition or clearance of a respiratory virus.

**Figure 8.**
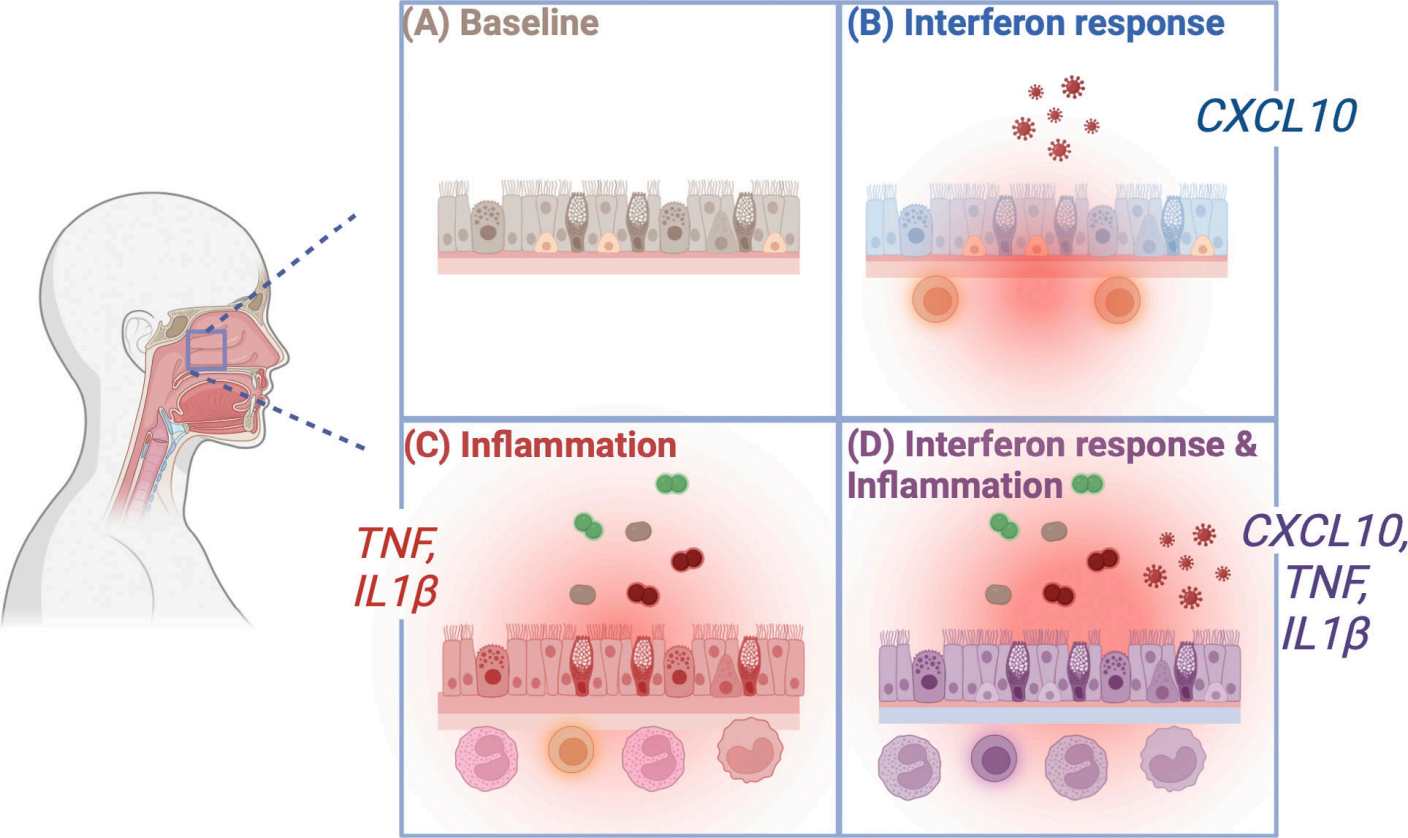
**Proposed model.** The presence of respiratory viruses and bacterial pathobionts drives distinct heightened immune phenotypes in children. Relative to virus-negative, pathobiont-negative subjects (A), mucosal respiratory viruses drive the interferon response (measured here as elevation of CXCL10), which results in epithelial expression of ISGs, including cell-intrinsic antiviral effectors and chemokines that recruit T cells and other leukocytes involved in innate and adaptive antiviral immunity (B). The presence of bacterial pathobionts that frequently colonize young children leads to a proinflammatory phenotype consisting of TNF and IL-1β secretion and enhanced recruitment of neutrophils and other leukocytes involved in mucosal containment of bacterial infection (C). Viral infection combined with bacterial pathobiont colonization leads to a combined immune phenotype with heightened interferon responses, proinflammatory cytokine secretion, and recruitment of leukocytes to the nasal mucosa (D).

In sum, by pairing comprehensive qPCR for respiratory viruses and bacterial pathobionts with nasal cytokine detection and RNA-seq data, this study identifies cytokine biomarkers of distinct virus-, pathobiont-, and coinfection-associated innate immune activation states. Our results support a model in which a high burden of respiratory viruses and bacterial pathobionts drive distinct patterns of heightened nasal mucosal immunity in children (Fig. 8).

## Discussion

Innate immune defenses at the site of infection are critically important in limiting the replication of respiratory viruses and promoting effective adaptive immune responses, prompting interest in understanding what regulates the set point of these defenses. Recent work during the SARS-CoV-2 pandemic reported that children, with or without SARS-CoV-2 infection, have heightened innate immune activation in the nasal mucosa compared with adults, including heightened activation of the interferon response, enhanced proinflammatory responses, and mucosal leukocyte infiltration (Loske et al., 2022; Mick et al., 2022; Pierce et al., 2021; Wimmers et al., 2023; Winkley et al., 2021; Yoshida et al., 2022). Using cytokine biomarkers of distinct patterns of mucosal inflammation, RNA-seq, and cytokine profiling, we show that heightened innate immunity in children is not a single phenotype but rather a spectrum of phenotypes (Fig. 8).

By comparing the pattern of mucosal inflammation with the presence and load of viruses and bacterial pathobionts in the same sample, we identified drivers and biomarkers of this spectrum of phenotypes. We show a clear relationship between viral presence and abundance in the upper respiratory tract and activation of the mucosal interferon response. We also find a strong association between bacterial pathobiont burden and activation of proinflammatory nasal mucosal responses, including production of IL-1β and TNF and neutrophilic inflammation. Using paired longitudinal samples from healthy 1-year-olds, we also showed that heightened mucosal activity of antiviral and proinflammatory responses is a dynamic phenotype, with innate immune activity changing concomitantly with the acquisition or clearance of a respiratory virus.

A key feature of our study was testing for 16 total respiratory viruses and three common bacterial pathobionts in samples from symptomatic and asymptomatic subjects. While the high prevalence of respiratory bacterial pathobionts in children is well known, the high frequency of respiratory viruses in children, including asymptomatic children, has only recently been appreciated due to the increasing use of symptom-agnostic viral PCR testing in research. Our results contribute to a growing literature showing that respiratory viruses are present at much higher rates than previously appreciated in the nasal mucosa of children. Additionally, even when a virus does not cause clinically significant disease, it may induce biologically significant ISG expression in the nasal mucosa that impacts immune responses to infections and/or vaccinations (Byington et al., 2015; Costa-Martins et al., 2021; Yahya et al., 2017; Yu et al., 2019).

While there are examples in the literature of viruses inducing proinflammatory responses and bacteria activating interferon responses, our results are striking in showing that there is a strong bias in patterns of mucosal innate immune activation induced by distinct microbial drivers. The presence and load of a respiratory virus were the only significant predictors of CXCL10, a biomarker of mucosal ISG expression. While both viruses and pathobionts were associated with proinflammatory cytokines, pathobiont positivity led to a greater increase in proinflammatory cytokines in cross-sectional samples. One factor that may reconcile these results with previous findings is that we observed high rates of viral-bacterial coinfections and a significant increase in virus-associated proinflammatory responses when bacterial coinfection was also present. Another factor is that viral load had a much wider dynamic range than bacterial load in paired samples (Fig. S5 C). The larger dynamic range suggests that viral load is the more important driver of dynamic changes in mucosal immune responses when both pathogen types are present. Our data from paired longitudinal samples from 1-year-olds further support this idea since biomarkers of both interferon and proinflammatory responses significantly increased during the acquisition of a viral infection. Notably, most subjects in this cohort also had bacterial pathobionts, so proinflammatory cytokine production reflects the combined effects of responses to viruses and coinfecting pathobionts.

Importantly, our results showing microbial drivers of heightened nasal innate immune states in children do not preclude a contribution of age-specific immunological developmental factors to heightened nasal innate immunity. In fact, our results suggest an interplay between the two. Recent work shows that major developmental changes occur in the leukocyte composition of the airway mucosa throughout early life, especially in children <5 years (Connors et al., 2023; Matsumoto et al., 2023). Our demonstration that viruses and bacterial pathobionts are highly prevalent and trigger mucosal cytokine and chemokine responses in this age group suggests that frequent host–pathogen interactions may be important drivers of early-life leukocyte dynamics in the respiratory tract. It is also possible that frequent infections lead to changes in the epithelium that persist between acute infections such as epigenetic changes, changes in the intracellular proteome, and/or changes in the cellular composition of the differentiated epithelium, any of which could contribute to heightened nasal innate immunity.

Mucosal phenotypes induced by immediate exposure to microbial drivers might not be expected to persist when cells are cultured ex vivo, but longer-lasting (e.g., epigenetic) microbe-induced changes might persist. Two recent studies showed evidence of enhanced interferon responses in cultured nasal epithelial cells from children compared with adults, including a high interferon-response goblet cell type unique to children seen during SARS-CoV-2 infection (Woodall et al., 2024; Zhu et al., 2022). Another study compared airway epithelial cells of children and adults using laser capture-microdissection of biopsies, airway basal cells sorted directly from biopsies, and proliferating primary basal epithelial cells in culture (Maughan et al., 2022). In biopsies and sorted basal cells, which captured the transcriptome at the time of sampling, interferon response pathways were elevated in children compared with adults, but this phenotype was not seen after in vitro culture. This result is consistent with the role of an interplay between environmental and cell-intrinsic factors in promoting heightened respiratory mucosal innate immune responses in children. Other age-related cell-intrinsic factors could also influence responses to viral infections such as the reported higher proliferation rate of pediatric respiratory epithelial cells (Maughan et al., 2022).

Identifying drivers and biomarkers of heightened nasal innate immunity in children opens the door to addressing unanswered questions related to these phenotypes. First, one reason for the great interest in differences between pediatric and adult mucosal immune responses during the COVID-19 pandemic was the lower impact of SARS-CoV-2 in children compared with adults regarding disease severity, hospitalizations, and mortality (CDC COVID-19 Response Team, 2020; Shekerdemian et al., 2020). Did heightened mucosal innate immunity due to a higher burden of other mucosal infections protect children against SARS-CoV-2? This is possible—recent work suggests that stronger barrier immunity in the respiratory tract of children and adults was associated with viral containment at the mucosa and less systemic spread (Sposito et al., 2021; Yoshida et al., 2022). Studies by our group and others in differentiated human respiratory epithelial cultures, and animal models show that ISGs induced by other viruses can suppress replication of SARS-CoV-2 in simultaneous or sequential infections (Cheemarla et al., 2021, 2023b, Dee et al., 2021, 2023; Essaidi-Laziosi et al., 2022; Oishi et al., 2022; Di Pietro et al., 2024). However, viral coinfections with SARS-CoV-2 can also increase infection severity, especially in experimental coinfection models in which the individual infections cause tissue damage and severe disease (Achdout et al., 2021; Kim et al., 2022; Kinoshita et al., 2021; Pizzorno et al., 2022). While the outcome of viral coinfections depends on many factors including the viruses involved, relative timing, and host susceptibility, we propose that when the first infection is well-controlled by the mucosal interferon response, the effect is more likely to be protective such as in the setting of asymptomatic or resolving viral infections that are common in young children.

The potential role of bacterial pathobionts in shaping SARS-CoV-2 disease severity is less clear. For seasonal respiratory viruses, bacterial pathobionts are known to contribute to acute symptoms and can also cause impactful secondary bacterial infections (Bosch et al., 2017; Brealey et al., 2018; de Steenhuijsen Piters et al., 2022; Kloepfer et al., 2014; Rodrigues et al., 2013; Teo et al., 2015). For SARS-CoV-2, there is also evidence from experimental models that pre-existing proinflammatory responses in the lung can contribute to protection from SARS-CoV-2, but the effects of specific bacterial pathobionts are not well-established (Baker et al., 2024, *Preprint*). It is also possible that the inflammatory response to bacterial coinfection could both increase acute upper respiratory symptoms and protect from systemic infection by enhancing innate and/or adaptive immune responses.

Although there are many possible factors contributing to the difference in susceptibility to COVID-19 illness in adults compared with children, if heightened mucosal antiviral responses in children played a role, why are children more susceptible than adults to seasonal respiratory viruses? Our results suggest an explanation that requires considering differences between children and adults in both innate and adaptive immunity. Compared with young children, adults are more protected from seasonal viruses by adaptive immune responses due to many prior exposures; this is thought to be a major reason why seasonal respiratory viruses are more highly prevalent and impactful in children (Tregoning and Schwarze, 2010). However, in the setting of a novel emerging virus such as SARS-CoV-2, neither adults nor children had adaptive immunity at the start of the pandemic. Based on our results, we propose that frequent seasonal viral infections in children (i.e., due to less well-developed adaptive immunity) trigger heightened mucosal interferon responses relative to adults, and that in the setting of a novel emerging virus, this dynamic low-level stimulation of mucosal interferon provided some protection against SARS-CoV-2.

More broadly, this model suggests that frequent low pathogenicity viral infections may have an important underappreciated protective function in early life. For example, RVs are extremely common in young children and occasionally cause severe illness, but the vast majority of RV infections cause common colds or are asymptomatic (Byington et al., 2015; Jartti et al., 2008; Winther et al., 2006; Wolsk et al., 2016). Such infections could play an important role in protecting against severe viral illnesses in early life by triggering broadly protective mucosal interferon responses at a time when young children are encountering many new viruses for the first time and have minimal protection from adaptive immunity.

Our study has several limitations. First, we measured a targeted panel of cytokine biomarkers, viruses, and bacteria in a large sample set, complementing prior work that used detailed immunophenotyping by RNA-seq and single-cell sequencing albeit in smaller sample sets. Future studies pairing detailed immunophenotyping with sensitive pathogen detection methods and clinical data will provide further insights into the range of nasal immunophenotypes in children and their drivers. Second, most of the symptomatic children in this study did not have severe disease and were treated as outpatients with limited follow-up; therefore it was not possible to make detailed conclusions about how nasal immunophenotypes influenced disease symptoms or outcomes. It will be important to investigate how nasal immunophenotypes at the time of infection impact severity and outcomes in future work.

In sum, we identified respiratory viruses and bacterial pathobionts as key drivers of heightened nasal innate immunity in children. These results compel further study of how seasonal respiratory viruses and bacterial pathobionts impact SARS-CoV-2 pathogenesis and compel further exploration of how frequent mucosal respiratory infections impact pediatric immune responses in general, including responses to other viruses encountered in early life and other immunological challenges including mucosal vaccines.

## Materials and methods

### Preoperative and pediatric ED NP swab collection and inclusion criteria

Study cohorts are summarized in Table S8. In brief, cross-sectional analyses included NP swabs collected for SARS-CoV-2 testing in the YNHH ED from children with and without symptoms of acute respiratory infection or from outpatients being tested for SARS-CoV-2 prior to elective surgery. The analysis included all available samples from two timeframes: June 3–July 2, 2021 (176 samples, 176 subjects) and January 11–23, 2022 (291 samples, 286 subjects). Inclusion criteria required all samples to have high RNA quality based on the internal albumin control in the YNHH respiratory virus panel (albumin, CT < 33), which included 467/483 samples curated during these time frames.

In the January 2022 cohort, five patients were sampled twice: once during the initial presentation and a second swab prior to discharge. Reanalysis of all affected analyses excluding the second NP swab did not change the results (Figs. 2, 3, 4, 5, 6, S1, and S3; see Mendeley data [Watkins and Foxman, 2024]). For viral coinfection analysis, an additional set of 167 NP swabs collected from 167 subjects in August 2021 is included. These samples had been tested as part of patient care using a four-plex respiratory virus (SARS-CoV-2, Influenza A/B, RSV) suggesting all were from symptomatic infections. All samples underwent qPCR testing for 16 viruses and bacterial pathobionts as part of this study. The study protocol was reviewed and approved by the Yale Human Investigation Committee, an institutional review board (IRB protocol #2000027656) and was determined to not require specific patient consent due to the use of residual samples and deidentification of subjects for reporting.

### Paired NP swab collection and inclusion criteria

Healthy children presenting to a pediatric practice in New Haven, CT for 1-year-old well-child visits were recruited for paired NP swab collection. Parents or legal guardians of children provided written informed consent before providing samples. Following consent, an NP swab was collected from children at the time of the 1-year-old well visit. Study participants were asked to return to the practice 7–14 days later for a second NP swab collection. NP swabs were subject to the same analysis and met the same inclusion criteria (albumin Ct < 33) as those collected from preoperative screening and pediatric ED visits. This study protocol was reviewed and approved by the Yale Human Investigation Committee, an institutional review board, and included parent/guardian informed consent (IRB protocol #2000030690).

### Chart review for symptom designation and admission comorbidities

To assign symptoms to subjects in the June/July 2021 and January 2022 cohorts, we extracted deidentified ICD-10 codes and performed manual chart reviews as follows. Patients were considered symptomatic if the ICD-10 code associated with presentation to the ED was consistent with acute respiratory illness (Table S1). Where ICD-10 codes for acute, symptomatic presentations were ambiguous, a manual chart review by a clinical reviewer blinded to bacterial, viral, and immune biomarker status was conducted for determination of clinical syndromic category. In these cases, patients were considered symptomatic if encounter notes included congestion, rhinorrhea, cough, sneezing, respiratory distress, increased respiratory effort, sore throat, adventitious breath sounds, or gastrointestinal symptoms accompanied by fever. If a known noninfectious cause was determined to be responsible for symptoms, patients were categorized as asymptomatic for respiratory infection symptoms.

### Cytokine measurements

Viral transport media was thawed on ice before measurement of cytokines and subsequently aliquoted and stored at −80°C for use in other experiments. Protein concentration in viral transport media was measured for human CXCL10, IL-1β, and TNF using the Simple Plex Ella platform (Protein Simple). One sample in the <5 years group was excluded from IL-1β and TNF analysis due to limited viral transport media.

### Clinical virology testing

NP swabs were placed in viral transport media (BD Universal Viral Transport Medium) immediately upon collection for clinical SARS-CoV-2 testing. Viral transport media from NP swabs was aliquoted and stored at −80°C for further analyses within three days of clinical testing. For virology testing, 200 µl of viral transport media were used for total nucleic acid extraction using the NUCLISENS easyMAG platform (BioMérieux). Extracted nucleic acid was tested using a 15-virus panel as described previously (Table S7) (Cheemarla et al., 2023a). SARS-CoV-2 testing was completed using four clinical testing platforms (Table S7). If the clinical testing platform did not report a Ct value in the medical record, we performed SARS-CoV-2 RT-qPCR manually using the CDC N1 gene assay as previously described (Cheemarla et al., 2021). Ct values from different SARS-CoV-2 assays were normalized using interpolation of the linear regression between samples with known E gene Ct values and known Ct values for other SARS-CoV-2 genes, which showed a strong correlation regardless of gene target (R^2^ > 0.98 for all pairs tested; Mendeley data [Watkins and Foxman, 2024]).

### Pathobiont RT-qPCR

All samples meeting inclusion criteria with sufficient residual sample were tested for *M. catarrhalis*, *H. influenzae*, and *S. pneumoniae*. 2 µl of total nucleic acid extracted during clinical testing was used for each pathobiont RT-qPCR assay. Luna Universal Probe qPCR Master Mix or AgPath-ID One-Step RT-PCR master mix (Applied Biosystems) was used with commercially available Taqman qPCR assays designed for each pathobiont (Thermo Fisher Scientific). Thermocycler conditions were set according to the manufacturer’s instructions. Ct results were quality-checked to reflect exponential, specific amplification of target pathobionts.

### Statistical analysis and data visualization

GraphPad Prism (10.1.2) was used for statistical analysis and data visualization for portions of Figs. 1, 2, 3, 5, 6, 7, S1, S3, and S5 with the statistical tests indicated in figure legends. For portions of Figs. 2, 3, 4, 5, 6, 7, S2, S4, and S5, RStudio (version 4.3.1) was used for data visualization and statistical analysis as indicated. Data curation and preparation were conducted through the use of tidyverse (version 2.0.0) and lubridate (version 1.9.2) R packages, while visualization was assisted by the ggplot2 (version 3.4.4) package (Grolemond and Wickman, 2011; Wickham, 2011; Wickham et al., 2019). Fig. 7 A and Fig. 8 are made using BioRender. For Table S1, the Mann–Whitney test was used to compare age distributions between virus and pathobiont categories. For the remaining demographics categories, chi-square values are calculated using RStudio; demographic categories that contain “NA/Not Disclosed” as a group do not include this group in chi-square calculations. Analysis of sex as a biological is available in the Mendeley data (Watkins and Foxman, 2024).

### Mediation analysis

For mediation analysis, we considered two different viral load categories. First, for all virus-positive individuals (*n* = 106), we considered the viral load (40-Ct) of the infecting virus for single infections and the highest viral load (i.e., minimum Ct value) for individuals identified with coinfections. Second, we focused on individuals with a SARS-CoV-2 single infection (*n* = 57) where only the viral load of SARS-CoV-2 was considered. We followed Baron and Kenny’s causal step approach using linear modeling of the effect (Baron and Kenny, 1986). Pre-requisites for indicating the appropriateness of the mediation analysis were confirmed by testing for a significant direct effect from the independent variable (IV; i.e., age) toward the dependent variable (DV; i.e., log_10_-transformed CXCL10) and a significant effect of the IV on the mediator (i.e., viral load). The mediation effect is then tested by considering the combined effect of the mediator and IV on the DV. In case the significant effect of the IV on the DV is reduced by the inclusion of the mediator, we can positively confirm the mediation. Lastly, casual mediation effects were computed for 1,000 bootstrapped samples and the 95% confidence interval was reported. The analysis was performed using RStudio (4.3.1) using the stats package for the linear modeling and mediation 4.5.0 for building the final mediation model and extracting information on the causal mediation effects (R Core Team, 2021; Tingley et al., 2014).

### Multiple linear regression analysis

To determine the effect of viral coinfection on the CXCL10 response, we combined data from all samples containing single infections and coinfections for each virus (i.e., SARS-CoV-2). We used RStudio (4.3.1) and the “glm” function (family = “gaussian”) to perform multiple linear regression analysis using the formula shown:

$$\log_{10}CXCL10∼\beta_{0}+\beta_{1}\left(40-Ct\right)+\beta_{2}\left(coinfect\right),$$

where *Ct* is the cycle threshold value of the respective virus being analyzed, and *coinfect* is a binary variable with 0 representing the absence of a coinfecting virus and 1 representing the presence of a coinfecting virus.

For Fig. 3 A, Fig. 6 D, Fig. S4, and Fig. S5 B, a multiple linear regression model approach was used to quantify variable associations with CXCL10, IL-1β, and TNF. A representative formula is shown below:

$$\log_{10}\left(cytokine\right)∼\beta_{0}+\beta_{1}\left(40-ViralCt\right)+\beta_{2}\left(40-PathobiontCt\right).$$

All multiple linear regression models were checked for goodness-of-fit using the “DHARMa” package (version 0.4.6) (Hartig, 2022). All models are subject to Kolmogorov–Smirnov tests, dispersion tests, and outlier tests. If the model leads to a significant P value for any of these tests, it does not meet sufficient goodness-of-fit, it is excluded from the results as indicated. Model outputs and goodness-of-fit testing are available in the Mendeley data (Watkins and Foxman, 2024).

### RNA-seq analysis of GSE172274

RNA-seq analysis of samples deposited in the NIH Gene Expression Omnibus (GEO) database under the accession number GSE172274 was carried out using Partek Flow software (version 10.0) for human transcripts (Pierce et al., 2021). Samples were aligned to the GRCh38 human genome using Bowtie 2 and quantified using Ensembl Transcripts Release 104 (Cunningham et al., 2022; Langmead and Salzberg, 2012). Counts were normalized as counts per million (CPM). The analysis focused on 50 genes found to be upregulated and differentially expressed in nasal epithelial cells during the antiviral response from a previous dataset (Landry and Foxman, 2018). Normalized counts were converted to Z-scores by subtracting the mean normalized expression of each gene from each sample’s normalized expression and dividing by the standard deviation of the gene’s expression across all samples. Finally, the ISG score was calculated by averaging the Z-scores for the 50 listed ISGs for each sample. Metagenomics analysis of samples in GSE172274 was analyzed using the Chan Zuckerberg CZID metagenomics pipeline as previously described (Kalantar et al., 2020). Non-SARS-CoV-2 virus reads were considered positive if above 10 rPM and were manually checked for sufficient genome coverage to rule out sequencing artifacts/contaminants. All samples in this dataset tested positive for SARS-CoV-2 by clinical testing as described previously (Pierce et al., 2021). Samples below the limit of detection for SARS-CoV-2 using metagenomics are set to the minimum nonzero SARS-CoV-2 read count detected in the dataset for visualization.

### RNA-seq analysis of phs002442.v1.p1

RNA-seq analysis was performed on datasets available on the NIH Database of Genotypes and Phenotypes (dbGaP) under accession code phs002442.v1.p1 as previously described (Cheemarla et al., 2023a). DEGs (FDR < 0.05, |Log_2_FC| >1) were identified using DESeq2 (version 1.22.1) (*n* = 3841). DEGs were converted to Z-scores, separated into two groups using hierarchical clustering, and visualized using RStudio (4.3.1) and the pheatmap package (1.0.12). GSEA was performed comparing RV-positive pathobiont-high to RV-positive pathobiont-low samples (Mootha et al., 2003; Subramanian et al., 2005). The gene set C5.GO:BP.v2023 was used as a reference and 1,000 permutations were run using the “gene_set” permutation setting and the timestamp = 1705948484560 (Liberzon et al., 2011). QIAGEN IPA (QIAGEN Digital Insights) was carried out using DEGs generated from comparing RV-positive/pathobiont-high and RV-positive/pathobiont-low samples (Krämer et al., 2014). Analysis of upstream regulators of DEGs listed in the results section was filtered on cytokines only.

### Proteomics analysis

This study includes a new analysis of proteomics results from our previous work to identify cytokines that were upregulated in RV-positive/pathobiont-high samples compared with RV-positive/pathobiont-low samples (Cheemarla et al., 2023a). Qlucore Omics Explorer was used to identify differentially expressed cytokines. Cytokines with <1 pg/ml expression across all samples were excluded and the remaining cytokine concentrations were log_2_-transformed and converted to Z-scores. Finally, two-group (*t* test) comparison between RV-positive/pathobiont-high and RV-positive/pathobiont-low samples was used to narrow down to the top 10 upregulated differentially expressed cytokines. The cytokine heatmap was generated using RStudio (4.3.1) and the pheatmap package (1.0.12) and displayed in increasing q-value.

### Online supplemental material

Fig. S1 shows viral load and pathobiont load by microbe detection status. Fig. S2 shows multiple linear regression results of CXCL10 vs. viral load in single- and co-infections. Fig. S3. shows IL-1β and TNF for RV^+^ and SARS-CoV-2 samples with and without pathobionts in children <5 years. Fig. S4 shows multiple linear regression results for CXCL10, IL-1β, and TNF using viral load, pathobiont load, and age as predictors. Fig. S5 shows virus and pathobiont test statistics and changes in load across sample pairs. Table S1 shows an overview of virus and pathobiont test results by patient demographics. Table S2 shows ICD-10 codes associated with symptomatic and asymptomatic designations and selected codes subject to further chart review. Table S3 shows COVID-19^+^ patient admissions and symptoms associated with admission. Table S4 shows detailed viral test results for each sample cohort by collection period and age group. Table S5 shows bacterial pathobiont test results for each sample cohort by collection period and age group. Table S6 shows sample counts for each age range depicted in Fig. 2, G and H. Table S7 shows viruses included in the YNHH Clinical Virology PCR panel and SARS-CoV-2 platforms used in this study. Table S8 shows an overview of sample cohorts analyzed in main and supplemental figures.

## Supplementary Material

Table S1 shows overview of virus and pathobiont test results by patient demographics.

Table S2 shows ICD-10 codes associated with symptomatic and asymptomatic designations and selected codes subject to further chart review.

Table S3 shows COVID-19^+^ patient admissions and symptoms associated with admission.

Table S4 shows detailed viral test results for each sample cohort by collection period and age group.

Table S5 shows bacterial pathobiont test results for each sample cohort by collection period and age group.

Table S6 shows sample counts for each age range depicted in Fig. 2, G and H.

Table S7 shows viruses included in the YNHH Clinical Virology test panel and SARS-CoV-2 platforms used in this study.

Table S8 shows an overview of sample cohorts analyzed in main and supplemental figures.

## Data Availability

The original data and code supporting this study have been deposited in Mendeley Data at https://doi.org/10.17632/g8ckr9zxbx.1 (Watkins and Foxman, 2024). Primary diagnosis (ICD-10 text) and patient demographics associated with each study code are omitted in the extended data due to patient privacy concerns. This paper also includes an analysis of existing, publicly available data published prior to this analysis. These are available on the NCBI Gene Expression Omnibus repository under the accession code GSE172274 and on the NCBI dbGaP under the accession code phs002442.v1.p1.
